# Efficacy of Pam3CSK4 as a cross-species adjuvant for polysaccharide vaccines in humanized mouse and non-human primate models

**DOI:** 10.1038/s41467-026-74194-7

**Published:** 2026-06-12

**Authors:** Jamie E. Jennings-Gee, Alexis E. Adams-Sims, Karen M. Haas

**Affiliations:** Department of Microbiology and Immunology, Wake Forest University School of Medicine, Winston-Salem, NC USA

**Keywords:** Adjuvants, Vaccines, Translational immunology, Infectious diseases

## Abstract

Polysaccharide-based vaccines are essential for preventing bacterial infections, but their effectiveness is limited by weak antibody responses and lack of suitable adjuvants. TLR4 agonists enhance polysaccharide-specific antibody responses through B cell–intrinsic TLR4-MyD88 signaling in mice, but this mechanism is not conserved in primates, prompting the search for alternative MyD88-activating agonists. In vitro, the TLR1/2 agonist Pam3CSK4 synergizes with strong BCR crosslinking to enhance activation and antibody secretion by mouse and human B cells. In vivo, Pam3CSK4 in squalene emulsion increases protective pneumococcal polysaccharide–specific antibody responses in both immunocompetent and humanized mice. Although a dual TLR2/7 agonist shows strong in vitro activity, it fails to enhance polysaccharide-specific IgG responses in vivo, consistent with antagonism observed when Pam3CSK4 and TLR7 agonists are combined. In contrast, incorporating Pam3CSK4 into an adjuvant containing a TLR4 agonist, synthetic cord factor, and squalene emulsion further enhances memory B cell generation and protective antibody responses in mice and restores adjuvant activity in non-human primates, supporting Pam3CSK4-based formulations as promising adjuvants for polysaccharide vaccines.

## Abstract (web summary)

Polysaccharide vaccines are crucial to immunize against bacterial infections but require efficacious adjuvants to induce protective immune responses. Here the authors explore the use of Pam3CSK4 as an adjuvant for polysaccharide vaccines in mouse and non-human primate models.

## Introduction

Antibody (Ab) responses to bacterial capsules and other pathogen-associated polysaccharides are essential for host protection. Polysaccharide antigens (Ags) typically behave as T cell-independent type 2 (TI-2) Ags, which elicit rapid Ab responses primarily through innate-like B cell subsets, including marginal zone and B1 B cells^1–9^. While TI-2 Ags can induce Ab (predominantly IgM) responses independently of cognate T cell help, T cells may still contribute to class switching and IgG production through bystander activation and/or non-classical T cell help^10,11^. Given the superior effector functions of IgG—including FcγR-mediated phagocytosis and complement synergy^12–14^—enhancing IgG responses to polysaccharide vaccines remains a key goal.

Current polysaccharide vaccines are typically administered as native Ags without adjuvants or as protein-polysaccharide conjugates with adjuvants. Despite their success, conjugate vaccines are costly and complex to manufacture and distribute. Adjuvants that can boost IgG responses to native polysaccharides offer a promising alternative for cost-effective coverage and rapid deployment. Traditional adjuvants like alum fail to enhance primary TI-2 responses^15^, and co-administration of TLR agonists in mice is ineffective unless administration is delayed by several days^16–18^, which is impractical for clinical implementation. Clinical trials with IL-12, GM-CSF, QS-21, CpG, and others have similarly failed to improve IgG responses to native pneumococcal polysaccharides (PPS)^19^. Early work with Ribi, consisting of *Salmonella typhimurium* monophosphoryl lipid A (MPL) and mycobacterial cord factor (trehalose-6,6’-dimycolate) in squalene oil-in-water emulsion, increased primary PPS-specific Ab responses in mice^20,21^. We previously demonstrated a related, but less toxic adjuvant consisting of *Salmonella minnesota* MPL, synthetic cord factor analog (synthetic trehalose dicorynomycolate; sTDCM, or D-(+)-trehalose-6,6’-dibehenate; TDB), and squalene emulsion significantly augments Ab responses to native polysaccharides in mice, as demonstrated by significantly increased IgG production, memory formation, and Ab secretion following boosting^4,22–24^. Using a mouse model, we identified a critical role for MyD88-driven signaling in B cells for these effects^22^, and determined that B-1b and marginal zone B cells were major contributors to the increased Ab produced^4^. Finally, we showed that iNKT cells were critical for the adjuvant effects on increasing polysaccharide-specific IgG in mice^24^.

Despite its potency in mice, we found in the current study that the MPL-TDCM-squalene emulsion adjuvant failed to elicit polysaccharide-specific Ab responses in non-human primates. Our analysis revealed that MPL is responsible for the majority of the adjuvant’s effect in mice. This provided the rationale for studies aimed at identifying alternative MyD88-activating TLR agonists to co-stimulate polysaccharide Ag-activated human B cell Ab production. Herein, we describe the potential for distinct TLR agonists to activate and antagonize polysaccharide-specific B cell responses and identify Pam3CSK4 as a TLR agonist that significantly promotes polysaccharide-specific Ab production by mouse, non-human primate, and human B cells. These findings may be leveraged to develop strategies to improve polysaccharide vaccine responses in humans.

## Results

### MPL combined with squalene emulsion significantly increases Ab responses to PPS and other TI-2 Ags in mice and depends on B cell intrinsic TLR4-MyD88 signaling

**Fig. 1:**
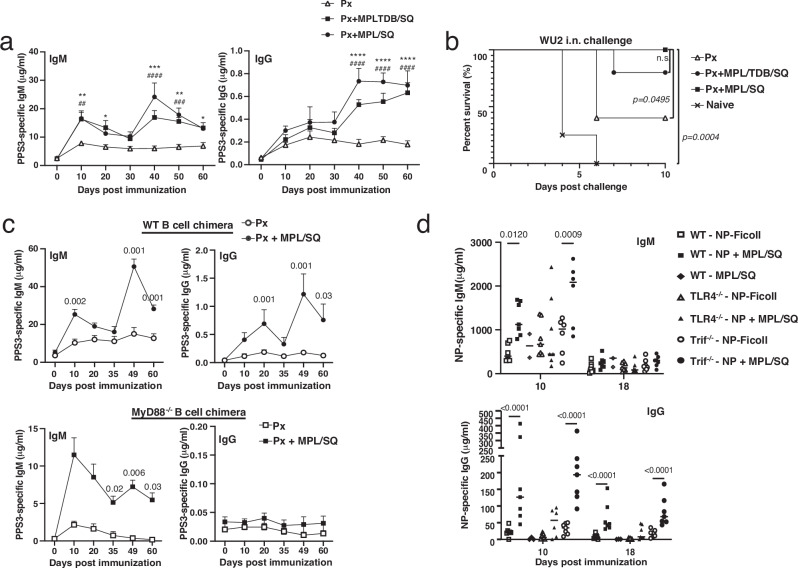
MPL + squalene emulsion functions as an adjuvant for polysaccharide-specific Ab responses and requires TLR4-MyD88 signaling on B cells. **a** PPS3-specific IgM and IgG levels in C57BL/6 wild type (WT) male mice immunized with Pneumovax23 (Px) alone or combined with MPL + squalene emulsion (SQ), or MPL + TDB + SQ i.m., with a boost on d30. Data are shown as means ± SEM. See Supplemental Fig. 1 for individual values. Statistical analysis performed using two-sided mixed effects model followed by Tukey’s multiple comparisons test (*indicates difference between Px and Px with MPL/TDB/SQ and #indicates differences between Px and MPL/SQ induced Ab levels, with *or #, *p* < 0.05; **or ##, *p* < 0.01; *** or ###, *p* < 0.001; **** or ####, *p* < 0.0001; *n* = 5 mice/group). **b** Survival following intranasal challenge with 10^7^ CFU serotype 3 *S. pneumoniae* (*n* = 5 immunized mice/group, *n* = 4 naïve mice; survival was analyzed using a two-sided log-rank (Mantel–Cox) test; exact P values are shown). Results representative of 2 independent experiments. **c** PPS3-specific IgM and IgG levels in male bone marrow chimeras reconstituted with male WT:muMt bone marrow or MyD88:muMt bone marrow (20:80). Data are shown as means ± SEM. See Supplemental Fig. 1 for individual values. Significant differences in primary (d0-20) and secondary (d35-50) responses were determined using two-sided repeated-measures ANOVA followed by Bonferroni’s multiple-comparisons test (*n* = 8 WT B cell chimeras and 4 MyD88 B cell chimeras per group). P values are indicated within graphs. **d** NP-specific IgM and IgG responses in female muMt mice reconstituted with 3×10^7^ female WT, TLR4^-/-^, or Trif^-/-^ B cells i.v. and immunized with 5 μg NP-Ficoll i.m. alone or mixed with MPL + SQ. Symbols represent data from individual mice, with horizontal lines indicating mean values. Statistical differences between groups of WT or Trif^-/-^ mice that were vaccinated with NP-Ficoll alone versus NP-Ficoll + MPL + SQ (n = 7 mice/group) were assessed by two-sided repeated-measures ANOVA followed by Bonferroni’s multiple-comparisons test. Exact p values are indicated above the relevant comparisons.

Our previous work demonstrated that a combination of MPL, TDCM (or TDB), and squalene emulsion significantly increases primary and secondary Ab responses to pneumococcal polysaccharide (PPS) and other TI-2 Ags, including haptenated Ficoll. This effect was strongly dependent on TLR4 and B-cell-expressed MyD88, with limited dependency on Trif signaling^22^. Given the strong dependency on TLR4 and B-cell-expressed MyD88, we investigated the extent to which MPL + squalene emulsion alone could function as an adjuvant for TI-2 Ab responses. We found MPL + squalene emulsion significantly increased PPS3-specific IgM and IgG responses to Pneumovax23 (Fig. 1a, Supplemental Fig. 1a) and significantly increased protection against intranasal challenge with 10^7^ CFU of serotype 3 *Streptococcus pneumoniae* (WU2) (Fig. 1b) in a manner similar to MPL + TDB + squalene emulsion. Consistent with our previous findings for MPL + TDCM + squalene emulsion^4,22,24^, MPL + squalene-induced increases in PPS3-specific IgG levels depended on B cell-expressed MyD88, as evidenced by the lack of adjuvant effect in bone marrow chimera mice reconstituted with MyD88-deficient B cells relative to chimeras reconstituted with WT B cells (Fig. 1c, Supplemental Fig. 1b), although PPS3-specific IgM responses were nonetheless increased during primary responses in MyD88-deficient B cell chimeras, but limited boosting was observed relative to WT chimeras. Notably, MPL + squalene significantly increased IgM and IgG responses to NP-Ficoll in B cell-deficient muMt mice reconstituted with B cells from WT and Trif^-/-^ mice, but not TLR4^-/-^ mice (Fig. 1d). Taken together, these data support that MPL combined with squalene functions as an adjuvant for TI-2 Ab responses, with effects dependent on B cell-expressed TLR4 and MyD88.

### The MPL-based adjuvant augments PPS Ab responses in aged mice but not NHP

**Fig. 2:**
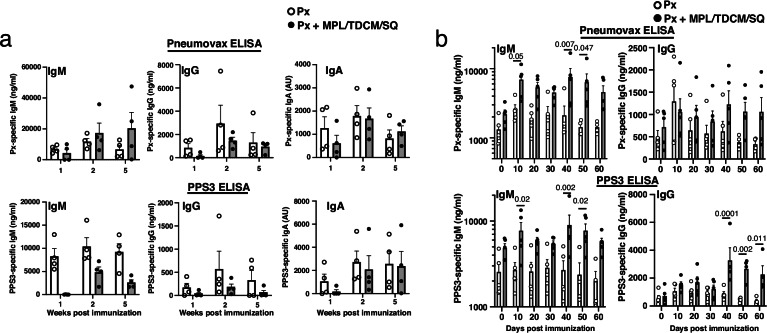
MPL + TDCM + squalene emulsion does not increase PPS-specific Ab responses in mature African Green monkeys but does so in aged mice. **a** Whole Pneumovax (Px)- and PPS3-specific IgM, IgG, and IgA responses in 17-20 year old female AGM at 1, 2, and 5 weeks post-vaccination (i.m.) with Pneumovax23 containing 12.5 μg each PPS alone or mixed with MPL + TDCM + squalene emulsion (SQ). Individual baseline Ag-specific Ab values were subtracted from week 1, 2, and 5 Ab values to determine vaccine-induced increases in PPS-specific IgM, IgG, and IgA levels. Individual data points are shown for each animal (*n* = 4 AGM/group) with mean values represented by bar height and error bars showing SEM. Note: IgA levels are expressed as arbitrary units (AU) due to the type of standard used (see methods). **b** Px- and PPS3-specific IgM and IgG responses in 20-24 month-old female C57BL/6 mice immunized (i.m.) with Pneumovax23 containing 0.125 μg each PPS alone or mixed with MPL + TDCM + SQ on d0 and 30 (*n* = 5 mice/group). Mean values are depicted by bar height (±SEM). Symbols represent data from individual monkeys (**a**) or mice (**b**), with bar height indicating mean values and error bars indicating SEM. Differences in Ab responses between groups were determined using two-sided repeated measures ANOVA with post-hoc Bonferroni’s multiple comparisons analysis. Significant differences are indicated by exact p values shown above bars.

Immunization of adult female African green monkeys (AGM) with Pneumovax23 alone or with MPL + TDCM+squalene emulsion adjuvant did not yield significant differences in whole Pneumovax23- or PPS3-specific IgM, IgG, or IgA levels between weeks 1 and 5 post immunization (Fig. 2a). We failed to find increases in Ab responses to PPS-4, −14, −19F, or −23F (Supplemental Fig. 2). The AGM used were 17-20 years old, which may be considered the equivalent of 50-65 year old humans^25^. We think it is unlikely the lack of effect in NHP was attributable to advanced age, as the adjuvant significantly increased Pneumovax23- and PPS3-specific IgM levels during primary responses in 20–24 month old female C57BL/6 mice, which approximate 60–70-year-old humans (Fig. 2b). Significant increases in Pneumovax- and PPS3-specific IgG were also observed in aged mice following boosting. Thus, the MPL-squalene emulsion adjuvant significantly increased protective pneumococcal capsule-specific Abs in mice, including aged mice, through a mechanism requiring TLR4-MyD88 signaling on B cells, but did not significantly increase polysaccharide-specific Ab responses in NHP.

### Evaluation of TLR agonists for co-activating human B cells in the context of BCR crosslinking

Given the requirement for B cell-specific TLR4 expression in supporting MPL-based adjuvant effects, the lack of effect in NHP, and the negligible expression of TLR4 on human versus mouse B cells^26,27^, we sought to identify other MyD88-activating TLR agonists that would potentiate increased TI-2 Ag-specific B cell activation and thereby promote increased primary and secondary Ab responses to polysaccharide Ags in humans. To do this, we stimulated human PBMC and CD19^+^-purified B cells with 1) alternative MyD88-activating TLR agonists, 2) strong BCR crosslinking using biotinylated anti-mouse Ig (H + L) F(ab’)_2_ Abs with streptavidin, or 3) both, and measured IgM-, IgG-, and IgA-secreting cells via ELISpot analysis 5 days later. Our goal was to identify agonists that stimulate little to moderate Ab production on their own but synergize with BCR signaling to significantly augment Ab production.

Flow cytometric analysis was performed on cultures of stimulated PBMCs isolated from four donors to evaluate the TLR agonist's co-stimulatory potential. BCR crosslinking alone significantly increased the percentage of B cells among PBMC relative to media or streptavidin-only wells after 5 days of culture (Supplemental Fig. 3). Addition of Pam3CSK4 (TLR1/2), FSL1 (TLR2/6), and CpG (TLR9) significantly increased the percentage of CD20^+^ cells in culture relative to both TLR agonist and BCR crosslinking alone. R837 (TLR7), R848 (TLR7/8), adilipoline (TLR2/7), and flagellin (TLR5) showed a similar trend for three out of four donors, whereas Addavax (squalene emulsion) had no effect. Similar trends were observed for the total number of B cells that had undergone CFSE-marked division and the overall number of IgG^+^ B cells following 5 days of culture, although only Pam3CSK4 + αIg cultures showed yields that were significantly increased over either stimulus alone across all four donors (Supplemental Fig. 3). Thus, although donor-to-donor variation was observed, all TLR agonists generally increased B cell numbers in PBMC cultures with BCR crosslinking, with Pam3CSK4 having the most consistent effect among donors.

**Fig. 3:**
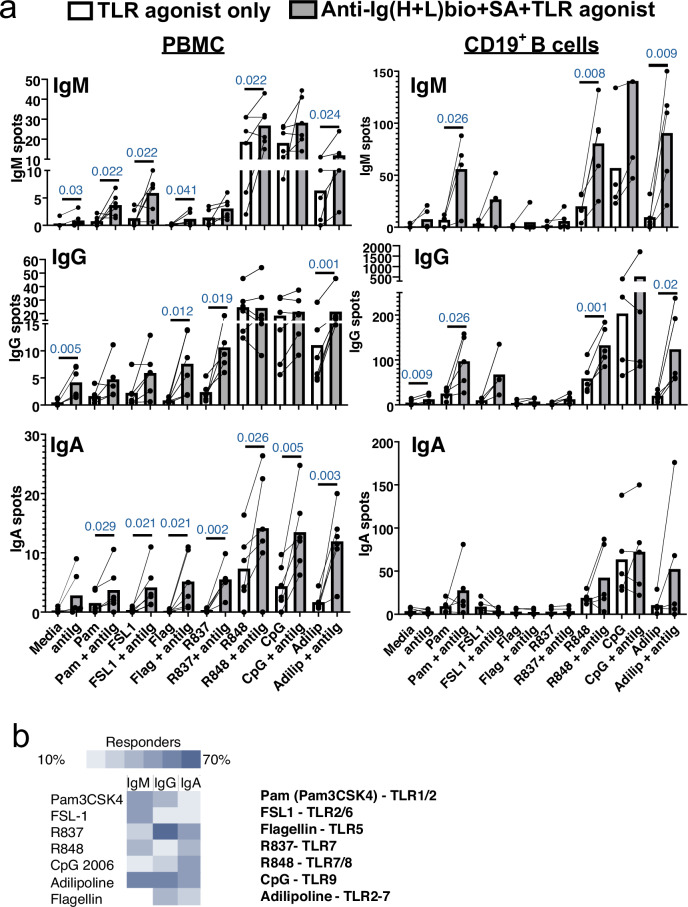
Activation of human B cell Ab secretion by TLR agonists and strong BCR crosslinking. **a** Human PBMC (10^5^/well) or CD19-selected B cells (10^4^/well) were cultured for 5 days in rhuIL-2 (10 ng/ml)-supplemented media, Pam3CSK4, FSL1, flagellin, R837, R848, adilipoline, or CpG2006 (1–2 μg/ml) alone or in combination with 1 μg/ml biotinylated goat anti-human Ig (H + L) F(ab’)_2_ plus 5 μg/ml streptavidin. On day 5, cells were transferred into ELISPOT plates and cultured for an additional 16 h, with total IgM, IgG, and IgA spots detected. Results shown for individual donors, with each symbol representing ASC yield for individual donors (*n* = 6 donors for PBMC stimulation, *n* = 5 donors for purified CD19^+^ B cell stimulation). Mean values are depicted by bar height. Exact p-values are shown for conditions for which TLR agonist plus BCR crosslinking are significantly different from values obtained for both BCR crosslinking alone and TLR agonist-only stimulation as determined by a one-tailed paired Student’s t-test, with lines connecting points from the same donor, and the p-value specifically shown for the TLR agonist only versus TLR agonist + BCR crosslinking condition. P values for the comparisons between the BCR crosslinking condition and the TLR agonist + BCR crosslinking condition can be found in the source data file. **b** Heatmap of ASC responses of 10 donors to TLR agonists plus BCR crosslinking versus TLR agonist alone, with positive responders for individual isotypes considered to have at least a two-fold increase in ASC numbers with TLR agonist plus BCR crosslinking relative to either stimulus alone.

Given the above results, we proceeded with ELISPOT analyses using all the TLR agonists described above. As shown in Fig. 3a, activation of BCR signaling alone in unfractionated PBMC or purified CD19^+^ B cell cultures induced modest numbers of ASCs compared to media alone. Pam3CSK4 had little effect on ASC yield when used alone, but significantly increased IgM and IgA ASC numbers when combined with BCR activation in PBMC cultures, and significantly increased both IgM and IgG ASCs in purified B cell cultures, indicating a role for direct B cell stimulation in co-activation. FSL-1 induced similar increases, albeit to a lesser extent. The TLR5 and TLR7 agonists, flagellin and R837, stimulated increased IgG and IgA ASC production in the presence of BCR crosslinking, but only in PBMC cultures, suggesting these effects required agonist activity on non-B cells^28^. Consistent with previous reports, when used alone, the TLR7/8 and TLR9 agonists, R848 and CpG, stimulated the greatest increases in IgM, IgG, and IgA ASCs in PBMC cultures relative to other agonists. Despite this, R848 combined with BCR crosslinking did not yield fold increases in ASC generation over that observed with Pam3CSK4 plus BCR crosslinking relative to Pam3CKS4 alone, and CpG-supported ASC generation was not greatly enhanced in B cells co-activated with BCR crosslinking. Finally, evaluation of a dual TLR2-TLR7 agonist, adilipoline, revealed its capacity to induce significant increases in IgM, IgG, and IgA ASCs in both PBMC and purified B cell cultures. Collectively, work evaluating subjects whose B cells in bulk PBMC cultures produced at least 2-fold greater ASC numbers with BCR crosslinking plus TLR agonist relative to either stimulus alone (*n* = 10 donors) revealed adilipoline, Pam3CSK4, and R837 supported the most consistent increases among donors, with adilipoline showing the most potent effect (Fig. 3b).

### TLR2 and TLR7 agonists used individually, but not in combination, increase protective PPS-specific IgG levels in mice in vivo

**Fig. 4:**
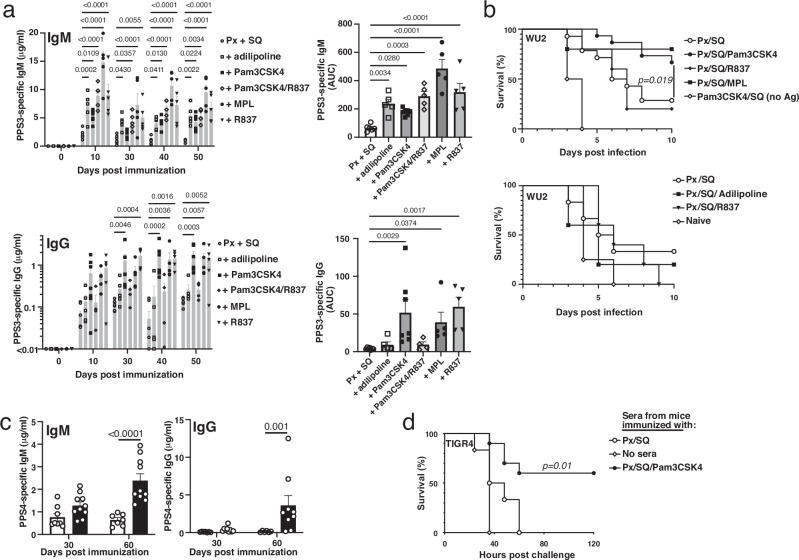
TLR2 and TLR7 agonists delivered in squalene emulsion function as adjuvants for PPS-specific Ab responses when used alone but not together in WT mice. **a** PPS3-specific IgM and IgG responses in WT male mice immunized i.m. with Pneumovax23 (Px) in SQ alone or combined with the indicated TLR agonists. Differences in responses between mice receiving TLR agonists versus mice immunized with Px-SQ alone were assessed by a two-sided mixed-effect model for repeated measures with Dunnett’s multiple comparisons analysis (*n* = 5–7 mice/group, Px+SQ, *n* = 7; Px+Pam, *n* = 6; all other groups *n* = 5) as well as area under the curve (AUC) analysis. Differences between groups for AUC were analyzed using a one-way ANOVA followed by Fisher’s LSD post-hoc test (two-sided). Mean values are depicted by bar height (±SEM), with data points shown for individual mice. Exact p-values comparing TLR agonist group results to the Px+SQ group are displayed above bars. **b** On day 75 post-immunization, mice were challenged i.n. with 10^7^ CFU *S. pneumoniae* strain WU2 and monitored for survival. Results from two pooled replicate experiments. The p-value shown indicates two-sided Log-rank (Mantel-Cox) test significance (*p* = 0.019) for the Px/SQ/Pam3CSK4 group (*n* = 15) versus Px-SQ group (*n* = 14 mice). **c** PPS4-specific IgM and IgG responses in mice from the immunization described in (**a**). Circles represent values from individual mice, and bar heights indicate the mean concentration ( ± SEM). Significant differences were assessed using a two-sided mixed-effects model followed by Bonferroni’s multiple comparisons test. Exact p-values are shown above bars. Results representative of two independent experiments. **d** Survival of CD19^-/-^ mice challenged i.p. with 1000 CFU serotype 4 strain TIGR4 co-delivered with 1 μl sera from mice immunized with Px/SQ/Pam3CSK4 (*n* = 11) versus Px-SQ (*n* = 6). Pooled results from two independent experiments. The p-value indicates a significant difference in survival between Px/SQ/Pam3CSK4 versus Px-SQ immunized mice as determined using a two-sided log-rank (Mantel-Cox) analysis.

Given the notable effects of adilipoline, Pam3CSK4, and R837 on the induction of ASC in the context of BCR crosslinking in human B cells in vitro, we assessed the potential of these agonists to function as adjuvants for PPS-specific Ab responses in vivo. For these experiments, wild-type C57BL/6 mice were immunized i.m. with Pneumovax23 mixed with Addavax (squalene emulsion) alone or further combined with TLR agonsts (MPL, Pam3CSK4, R837, adilipoline, or Pam3CSK4 plus R837). As shown in Fig. 4a, all TLR agonists significantly increased PPS3-specific IgM levels over Pneumovax23-squalene, although MPL generally yielded higher levels than other agonists based on area under the curve (AUC) analysis. However, when single agonists (Pam3CSK4, R837, and MPL) with squalene emulsion were used, they significantly increased PPS3-specific IgG levels to at least 10-fold higher levels than those produced in response to Pneumovax-squalene emulsion in control animals, whereas mice immunized with the dual agonist, adilipoline or the Pam3CSK4/R837 combination failed to produce appreciable increases in PPS3-specific IgG (Fig. 4a). Similar trends were found when pooled PPSs were used in ELISA coating, although measured responses were more variable among individual mice (Supplemental Fig. 4a). Flagellin similarly reduced Pam3CSK4 adjuvant-mediated increases in PPS-specific IgM and IgG levels (Supplemental Fig. 4b), suggesting antagonistic effects were not limited to TLR7 agonists.

Interestingly, in mouse splenocyte cultures containing V_H_B1-8 transgenic (Tg) B cells, adilipoline, Pam3CSK4, R837, and the Pam3CSK4/R837 combination drove NP-specific IgM production when used in combination with NP-Ficoll-mediated activation (Supplemental Fig. 4c). However, only Pam3CSK4 significantly increased NP-specific IgG production in NP-Ficoll-activated B cells (Supplemental Fig. 4c, d); moreover, R837 significantly suppressed Pam3CSK4-mediated increases in NP-specific IgG (Supplemental Fig. 4c). Similarly, Pam3CSK4-supported increases in anti-Ig-induced ASC in purified human B cell cultures were reduced when R837 was added (Supplemental Fig. 4e). Collectively, these data support TLR7 and TLR2 agonists function as adjuvants for polysaccharide Ags when used individually, but when combined, suppress the individual adjuvant effects on Ag-specific IgG responses.

In contrast to the TLR2-7 combination agonists which did not provide increased protection against lethal respiratory pneumococcal infection with serotype 3 pneumococcus over the non-adjuvanted vaccine alone, inclusion of Pam3CSK4-squalene emulsion in the pneumococcal vaccine significantly increased protection to a level similar to that when MPL-squalene emulsion was included in the vaccine (Fig. 4b). Notably, Pam3CSK4-squalene emulsion not only increased PPS-specific IgG3, but also IgG1, IgG2b, and IgG2c levels (Supplemental Fig. 4f), which have an increased capacity for driving FcγR-mediated phagocytosis. In addition to significantly increasing PPS3-specific protective Ab responses, Pam3CSK4 functioned to significantly increase the level of protective PPS4-specific Abs as evidenced by the significantly increased protection in passive sera transfer experiments with lethal systemic serotype 4 (TIGR4) challenge (Fig. 4c, d). Importantly, Pam3CSK4 had no effect on PPS Ab responses if squalene emulsion was not included in the vaccination (Supplemental Fig. 4g). Collectively, this data supports that the TLR1/2 agonist, Pam3CSK4, when combined with squalene emulsion, functions as a potent adjuvant promoting significantly increased production of PPS-specific Abs that provide protection against lethal pneumococcal infection relative to the non-adjuvanted vaccine in mice. However, when a TLR7 agonist is combined with Pam3CSK4, it no longer increases protective PPS-specific Ab responses.

### Pam3CSK4 plus squalene emulsion functions as an adjuvant for protective human B cell responses to pneumococcal polysaccharides

**Fig. 5:**
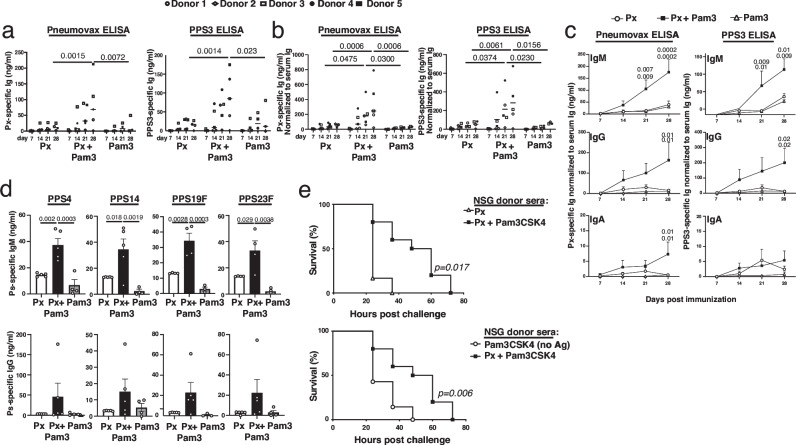
Pam3CSK4-squalene emulsion functions as an adjuvant to augment protective PPS-specific Ab responses in humanized NSG mice. NSG mice were reconstituted with human PBMCs and immunized the next day with Pneumovax (Px), Px + Pam3CSK4 + squalene emulsion (SQ), or Pam3CSK4 + SQ without antigen. **a** Px- and PPS3- specific Ig levels in mice reconstituted with cells from donors 1–5 (1–2, female; 3–5, male). Mice were sex-matched with the donor sex. **b** Ab concentrations in (**a**) were normalized to total serum Ig reconstitution levels for individual mice. Values for the five individual donors are indicated by distinct symbols. **c** Px- and PPS3-specific IgM, IgG, and IgA levels (±SEM) in NSG mice following immunization. Mean values are shown (±SEM). **d** PPS4-, 14-, 19F-, and 23F-specific IgM and IgG levels in reconstituted NSG mice on d21 post immunization, with values shown for individual donors, and mean values indicated by bar height (±SEM). In (**a–d**), exact p-values are shown in graphs to indicate significant differences (*p* < 0.05) between Px + Pam3CSK4 and Px only and adjuvant only mice (data were analyzed using a two-sided mixed effects model with Dunnett’s multiple comparisons test in (**a–c**) and using a one-way ANOVA in (**d**) with Dunnett’s multiple comparisons test (two-sided)). In (**c**), the top number represents the p value for the Px+Pam3CSK4-SQ vs. Px comparison, and the bottom number represents the p value for the Px+Pam3CSK4-SQ vs. adjuvant (Pam3CSK4) only comparison. Data are derived from five donors, except for the Pam3CSK4-only group, in which four donors were used. In (**a**), (**b**), and (**d**), symbols indicate values for individual donors. **e** Survival following passive transfer of d21 sera (equivalent to 3 μg of Ab) from huPBMC-reconstituted immunized NSG mice into CD19^-/-^ mice infected i.p. with 100 CFU WU2 serotype 3 *S. pneumoniae*. Results are pooled from repeated experiments. Log rank analysis results (two-sided) are indicated with significant differences indicated by the p values shown in the graphs (*n* = 10 mice in Px+Pam3CSK4 group, *n* = 7 mice in Px only group, and *n* = 6 mice in Pam3CSK4 only group).

Given our in vitro results with human B cells and our in vivo results in mice, we determined whether the Pam3CSK4/squalene-based adjuvant would increase PPS-specific Ab responses in humanized NSG mice. Fresh donor PBMCs were used to reconstitute NSG mice, with recipient mice subsequently immunized with the Pneumovax vaccine alone, vaccine plus Pam3CSK4 + squalene emulsion, or Pam3CSK4 + squalene emulsion without antigen. Successful reconstitution was confirmed by the presence of circulating Abs (Supplemental Fig. 4h). Reconstituted mice immunized with Pneumovax + Pam3CSK4 + squalene emulsion generated significantly higher levels of total Pneumovax23- and PPS3-specific Ig by 28 days post immunization (Fig. 5a). Upon normalizing PPS-specific Ab levels to the total circulating Ab levels for individual mice to account for differences in reconstitution, we found that mice immunized with the vaccine plus Pam3CSK4 + squalene emulsion had significant increases in Pneumovax23- and PPS3-specific immunoglobulins on both days 21 and 28 post immunization (Fig. 5b). Analysis of Ag-specific IgM, IgG, and IgA indicated the majority of these Abs belonged to IgM and IgG subclasses, with the adjuvant significantly increasing total PPS- and PPS3-specific IgM responses at both d21 and d28 and for IgG responses, at d28 relative to non-adjuvanted vaccine or the adjuvant alone (Fig. 5c). IgA responses were more variable; however, the adjuvanted vaccine significantly increased IgA responses to the PPS pool on d28. Analysis of Ab responses to other individual PPS included in the vaccine revealed similar trends, with the Pam3CSK4 adjuvant significantly increasing PPS4-, PPS14-, PPS19F-, and PPS23F-specific IgM responses relative to responses to the non-adjuvanted vaccine (Fig. 5d), and IgG responses in 3 to 4 out of 5 donors for PPS14, 19 F, and 23 F (Fig. 5d). Importantly, consistent with the increased PPS3-specific IgM and IgG in sera of mice immunized with vaccine plus Pam3CSK4-squalene emulsion, passive transfer of pooled d21 sera from these mice (equivalent to 3 μg total Ig) yielded significantly increased protection against lethal systemic serotype 3 *S. pneumoniae* infection in immunodeficient CD19^-/-^ mice relative to sera from reconstituted mice immunized with the vaccine or adjuvant alone (Fig. 5e). Thus, Pam3CSK4 combined with squalene emulsion functions as an adjuvant that significantly increases protective PPS-specific Ab responses by human B cells.

### Addition of Pam3CSK4 to the MPL/TDCM adjuvant further increases its potency for inducing protective polysaccharide-specific Ab levels with enhanced opsonophagocytic activity and supports functional memory B cell generation

**Fig. 6:**
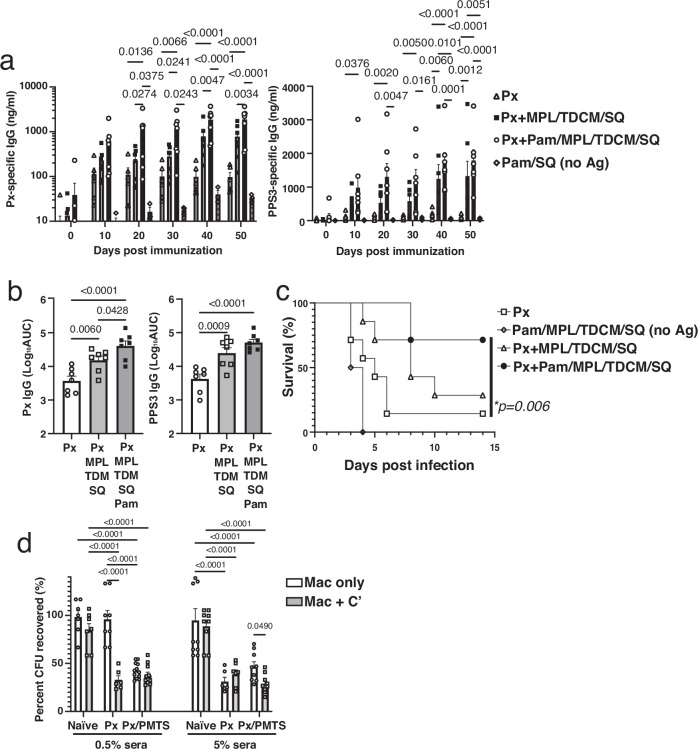
Combining Pam3CSK4 with MPL/TDCM/SQ adjuvant increases its potency for increasing protective PPS-specific IgG responses in WT mice. **a** Pneumovax23 (Px)- and PPS3-specific IgG responses in WT mice immunized i.m. with Px alone or Px+ MPL/TDCM/SQ, Px+ MPL/TDCM/SQ + Pam3CSK4, or Pam3CSK4 + SQ only (no antigen). All groups received the same immunization on d30. Mean values are shown (±SEM). Exact p values are shown, indicating significant differences as determined by two-way ANOVA followed by Tukey’s HSD (two-sided) post-hoc analysis (*n* = 7 female mice/group except for Pam3CSK4-SQ (no Ag) where *n* = 4). Results representative of three independent experiments. **b** Area under the curve (AUC) analysis for data in (**a**), with data points shown for the AUC calculated for individual mice. Bar height indicates mean AUC for each group (±SEM), with p values shown in graphs (one-way ANOVA, with significant differences followed by Fisher’s LSD post-hoc analysis for two-sided significance). **c** Mice immunized as described in A were challenged i.n. with 2×10^7^ CFU *S. pneumoniae* strain WU2 on d64 post immunization and monitored for survival. P-value shows two-sided Log rank analysis significance (*n* = 7/group). **d** Opsonophagocytosis assays performed with peritoneal macrophages (Mac) using 0.5 and 5% sera from naïve and Px-Pam-MPL/TDCM-squalene (PMTS) immune (d50) mice alone or with 25% rabbit complement (C’), with the percent *S. pneumoniae* CFU recovered indicated. Individual symbols indicate values for individual sera applied in OPA (0.5% naïve+Mac, *n* = 7; 0.5% naïve Mac+C’, *n* = 7; 0.5% Px+Mac, *n* = 8; Px+Mac+C’, *n* = 6; 0.5% Px+PMTS+Mac, *n* = 12; 0.5% Px+Mac+C’, *n* = 12; and 5% naïve+Mac, *n* = 9; 5% naïve Mac+C’, *n* = 9; 5% Px+Mac, *n* = 6; 5% Px+Mac+C’, *n* = 7; 5% Px+PMTS+Mac, *n* = 9; Px+Mac+C’, *n* = 9). Results were pooled from three independent assays. Mean values are indicated by bar height (±SEM). Exact p-values show significant differences between groups as determined using a two-sided two-way ANOVA followed by Fisher’s LSD post hoc test to identify specific pairwise differences. In (**a**), (**b**), and (**d**), symbols represent values obtained for individual mice.

Encouraged by the positive results for Pam3CSK4 on enhancing human B cell responses to PPS in vivo, we investigated whether other agonists could be combined with Pam3CSK4 to further increase protective PPS responses in immunocompetent C57BL/6 mice. We found that TDB, similar to TDCM in our original adjuvant formulation, further increased Ab responses when combined with Pam3CSK4 and squalene emulsion (Supplemental Fig. 4i). We therefore assessed whether combining Pam3CSK4 with the commercial adjuvant containing MPL/TDCM and squalene emulsion (Sigma Adjuvant System) that was originally administered along with Pneumovax23 to AGM with no effect (Fig. 2), improved PPS Ab responses in mice. As shown in Fig. 6, addition of Pam3CSK4 to this adjuvant significantly increased Pneumovax23- and PPS3-specific IgG responses in mice (Fig. 6a, b). Consistent with these Ab responses, mice that received this combination exhibited the highest level of protection against high dose lethal respiratory infection with serotype 3 *S. pneumoniae* (Fig. 6c). Using standard opsonophagocytosis assays^29,30^, we found that at 0.5% serum, only sera from adjuvanted mice mediated significant complement-independent macrophage clearance, while addition of complement facilitated macrophage opsonophagocytosis for non-adjuvanted immune sera (Fig. 6d). At 5% sera, complement further boosted macrophage-mediated clearance for sera from mice that had received adjuvant (Fig. 6d), pointing the potential for both Fcγ and complement receptor-mediated phagocytosis in immune sera from mice that had received the Pam3CSK4-based adjuvant. Consistent with this, Pam3CSK4-MPL/TDCM, as is the case for MPL/TDCM^22^, significantly increased production of PPS3-specific IgG3 and IgG2b (Supplemental Fig. 4j), which, together with IgM, synergize for optimal phagocytosis and clearance via stimulating both complement and Fcγ receptor-mediated signals^31–33^.

**Fig. 7:**
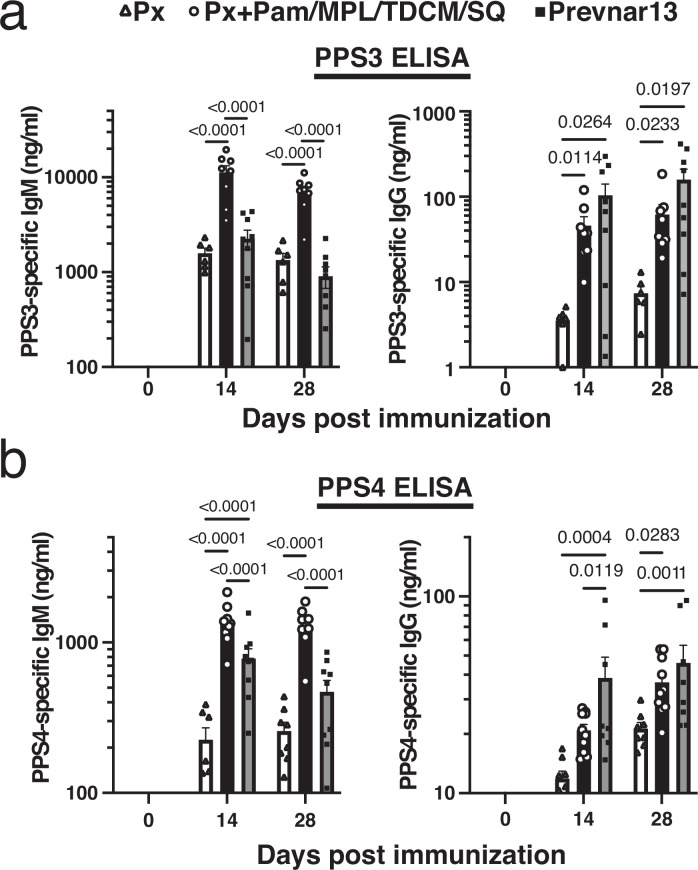
Pneumovax23 administered with Pam3CSK4-MPL/TDCM/SQ adjuvant yields significantly increased PPS-specific IgM and similar levels of IgG to Prevnar13. **a**,**b** Male mice were immunized with Pneumovax23 (Px) alone, Px+ Pam3CSK4-MPL/TDCM/SQ, or Prevnar13, with each dose containing 0.125 μg PPS3 and PPS4. PPS3 (**a**) and PPS4 (**b**)-specific IgM and IgG were measured by ELISAs (in **a**, Px, *n* = 6/group; Pam3/MPL/TDCM/SQ, *n* = 8; and Prevnar13, *n* = 9; in B, Px, *n* = 7/group; Pam3/MPL/TDCM/SQ, *n* = 9; and Prevnar13, *n* = 9). Results representative of two independent experiments. Mean values are indicated by bars (±SEM). Exact two-sided p values shown in the graphs indicate significant differences between groups as determined by two-way ANOVA significance followed by Fisher’s LSD post hoc test (two-sided) to identify specific pairwise differences.

Given the robust PPS-specific IgM and IgG responses produced when Pam3CSK4-MPL/TDCM-squalene is included with Pneumovax23, we examined how these Ab responses compared with those elicited by Prevnar13, which contains PPS-CRM197 protein conjugates adjuvanted with aluminum phosphate. As shown in Fig. 7a, Pneumovax23-Pam3CSK4-MPL/TDCM-squalene immunized mice produced 6-fold more PPS3-specific IgM compared to mice immunized with either Pneumovax23 alone or Prevnar13. Although both adjuvanted Pneumovax23 and Prevnar13 produced significantly more ( ~10-fold) PPS3-specific IgG than non-adjuvanted Pneumovax23, there was no difference between the level of IgG they induced. Similar results were found for PPS4-specific Ab responses (Fig. 7b). Thus, Pam3CSK4-MPL/TDCM-squalene adjuvanted Pneumovax23 produced comparable levels of PPS3- and PPS4-specific IgG and significantly more IgM than Prevnar13.

**Fig. 8:**
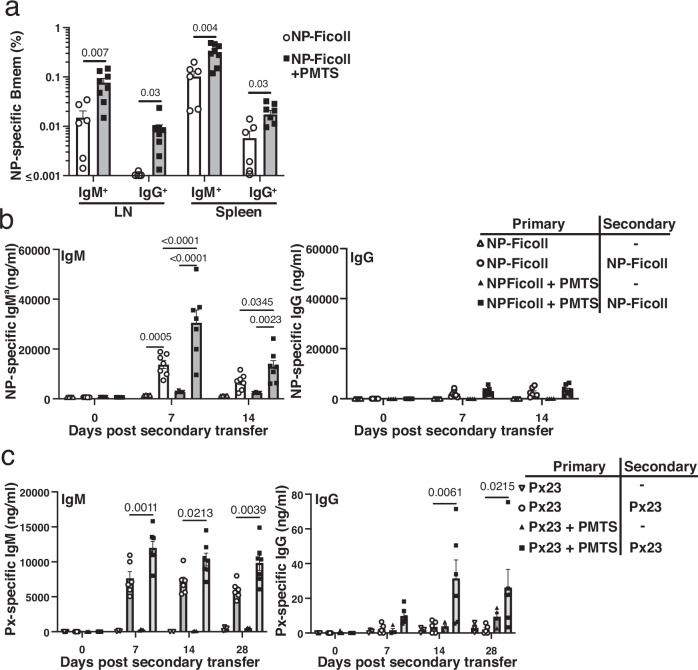
Pam3CSK4-MPL/TDCM/SQ adjuvant increases generation of functional memory B cells. **a**,**b** WT mice were reconstituted with CD43^-^CD45.1^+^V_H_B1-8 Tg B cells and immunized with 5 μg NP-Ficoll alone (*n* = 6 males) or with Pam3CSK4-MPL/TDCM/SQ (PMTS) adjuvant (*n* = 8 males) i.m. **a** Five weeks later, memory cells in the spleen and draining lymph nodes were assessed by flow cytometry. Mean frequencies (±SEM) are indicated by bar height, with symbols representing individual animals. Significant differences are indicated by the exact p-values shown in graphs as determined by a two-sided unpaired Student’s t-test. **b** Harvested splenic memory B cells were transferred into naïve WT male recipients. These recipients either remained naïve (*n* = 4) or were immunized with 5 μg NP-Ficoll i.m. (*n* = 7), with NP-specific IgM and IgG responses assessed. Mean values (±SEM) are indicated by bar height, with symbols representing Ab levels produced by individual animals. Significant differences are indicated by the exact p-values shown in graphs as determined by a two-sided unpaired Student’s t-test. **c** Splenic B cells from WT mice immunized with Pneumovax23 were adoptively transferred into naïve WT male recipients. These recipients either remained naïve (*n* = 4) or were immunized with Pneumovax23 i.m. (*n* = 6), with Pneumovax23-specific IgM and IgG responses assessed and mean values indicated (±SEM). Symbols indicate values for individual animals. Significant differences between boosted mice that received Px only priming versus Px+PMTS priming are indicated by p-values shown in graphs as determined by mixed-effect repeated-measures ANOVA with two-sided Bonferroni’s multiple comparisons test.

A key feature of successful vaccination is the generation of memory B cells that can rapidly produce Ab upon antigen re-exposure. We therefore investigated the potential for Pam3CSK4-MPL/TDCM-squalene to increase functional memory B cell formation using the tractable V_H_B1-8 Tg system. Similar to the effects observed with Pneumovax23, Pam3CSK4-MPL/TDCM-squalene significantly increased IgM and IgG responses to NP-Ficoll in WT mice reconstituted with resting CD43^-^V_H_B1-8 Tg B cells (Supplemental Fig. 5a). Endogenous NP-specific IgM^b^ responses were also significantly increased in recipient mice in response to adjuvant (Supplemental Fig. 5b). Analysis of resting non-Ab-producing CD138^-^ B cells in recipient mice 5 weeks post immunization, which represent memory cells based on our previous work^1,23^, revealed the adjuvant significantly increased frequencies of V_H_B1-8 Tg CD45.1^+^ CD138^-^ IgM^+^ and IgG^+^ NP-specific B cells in spleen by 3-fold and in the draining lymph node by 5-fold for CD138^-^IgM^+^ and 25-fold for CD138^-^IgG^+^ B cells relative to mice immunized with NP-Ficoll alone (Fig. 8a and Supplemental Fig. 5c). The V_H_B1-8 Tg NP-specific memory B cells expressed similarly elevated levels of CD80, PDL2, and CD73, with IgG^+^ memory B cells expressing the highest levels as we previously reported^1,23^, regardless of whether they received Ag with adjuvant (Supplemental Fig. 5d). However, splenic IgM^+^ memory B cells generated in the presence of adjuvant expressed significantly higher levels of CD11b and exhibited increased NP-APC binding, suggestive of possible innate B cell origin. Secondary transfers of these memory B cells from primary recipients into naïve secondary recipients demonstrated that NP-specific IgM recall responses to NP-Ficoll only (without adjuvant) were significantly increased for memory cells elicited in the presence of adjuvant (Fig. 8b), supporting that the adjuvant promotes development of functional memory cells, albeit predominantly IgM^+^ memory cells in the V_H_B1-8 Tg B cell system. We performed similar adoptive transfer experiments using WT mice immunized with Pneumovax23 alone or with Pam3CSK4-MPL/TDCM-squalene as donors. We similarly found that mice that received B cells from adjuvant-immunized donors produced significantly more IgM and IgG to Pneumovax23 (Fig. 8c) and PPS3, as well as more IgM to PPS4 (Supplemental Fig. 5e). Thus, Pam3CSK4-MPL/TDCM-squalene supports the development of functional memory B cells that have the capacity to respond to Ps Ag and produce Ab in the absence of adjuvant.

### Addition of Pam3CSK4 to the MPL/TDCM adjuvant restores adjuvant activity in NHP

**Fig. 9:**
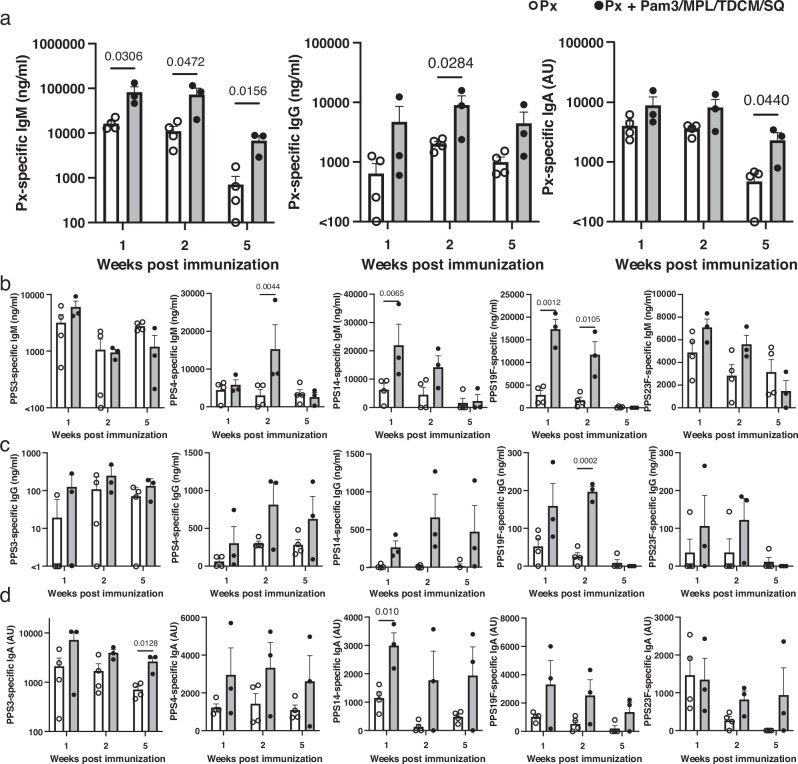
Addition of Pam3CSK4 to MPL/TDCM/SQ restores adjuvant activity for polysaccharide-specific B cell responses in NHP. **a–d** Px-specific IgM, IgG, and IgA (**a**) and PPS3-, 4-, 19F-, and 23F-specific IgM (**b**), IgG (**c**) and IgA (**d**) responses in 8–10 year old female AGM 1, 2, and 5 weeks post-vaccination (i.m.) with Pneumovax23 containing 12.5 ug each PPS alone or mixed with MPL/TDCM/SQ + Pam3CSK4 (*n* = 4/group). Mean values are indicated by bars (±SEM). Numbers above bars indicate exact p-values where significant differences in values were determined by a two-tailed unpaired Student’s t-test. Individual baseline Ag-specific Ab values were subtracted from week 1, 2, and 5 Ab values to determine vaccine-induced increases in PPS-specific IgM, IgG, and IgA levels. Circles represent values for individual animals.

Given that Pam3CSK4 further enhanced the response to MPL/TDCM-squalene emulsion in mice, we investigated whether inclusion of Pam3CSK4 in the MPL/TDCM-squalene emulsion adjuvant, which on its own was unable to increase PPS-specific Ab responses in AGM, would yield adjuvant effects. As shown in Fig. 9a, in contrast to results with MPL/TDCM-squalene emulsion (Fig. 2 and Supplemental Fig. 2), Pam3CSK4/MPL/TDCM-squalene emulsion significantly increased Pneumovax-specific IgM, IgG, and IgA responses (4- to 10-fold). Although variability in responses was evident among animals, significant increases in serotype-specific Abs, including PPS4- and PPS19F-specific IgM and IgG, were observed, along with significantly increased PPS3- and PPS19F-specific IgA levels (Fig. 9b–d) for animals that had received adjuvant. Specifically, we observed increases in IgM, IgG, and IgA responses to serotypes 4, 14, 19 F, and 23 F in 2/3 to 3/3 animals, and for serotype 3, increases in IgG (2/3) and IgA (3/3). Collectively, the restoration of MPL/TDCM-squalene emulsion adjuvant effects upon addition of Pam3CSK4 highlights its potential to function as an adjuvant for polysaccharide-based vaccines in primates, including humans, as supported by our humanized mouse data.

## Discussion

Polysaccharide Ags, such as those found in bacterial capsules, are critical targets for protective Ab responses, yet their T cell-independent nature, particularly in the absence of additional stimulatory signals, presents a challenge for vaccine design. TI-2 Ags primarily activate Ab production by innate-like B cell subsets, including marginal zone and B1 B cells, which rapidly produce IgM but produce modest IgG responses without additional signals^2,9,22,34^. Although IgM can confer protection, IgG is generally preferred in vaccine contexts due to its superior effector functions, including FcγR-mediated phagocytosis^12–14,35^. While protein-polysaccharide conjugate vaccines have successfully provided protection, especially in young children, the reliance on such complex vaccines poses a potential vulnerability. The lack of suitable adjuvants for co-administration with native polysaccharide vaccines represents an understudied area that could lead to the development of suitable alternative routes to protection. Herein, we demonstrate a critical role for TLR4/MyD88-mediated co-stimulation in polysaccharide-specific B cell activation, enabling a TLR4-activating adjuvant to increase protective polysaccharide-specific Ab levels in mice. Despite its potent effects in mice, we show the MPL-based adjuvant had little effect in NHP, thereby supporting the evaluation of alternative MyD88-activating TLR agonists for this purpose. Our in vitro results indicate that most MyD88-activating TLR agonists have B cell-stimulatory activity, either directly on human B cells or in concert with accessory cells present in PBMCs. In particular, Pam3CSK4 emerged as a potential alternative due to: 1) its ability to co-stimulate both human and mouse B cell activation, expansion, and ASC formation (including IgG production) in the presence of strong BCR crosslinking, and 2) its ability to significantly increase production of protective PPS-specific Abs in vivo in wild-type mice, PBMC-humanized NSG mice, and African green monkeys. Of significant interest, we also identified antagonistic activity when either TLR7 or TLR5 agonists were combined with Pam3CSK4, whereas the opposite was true when TLR1/2 and TLR4 agonists were paired. Collectively, our findings support the strategic use of TLR1/2 agonists for next-generation adjuvant design to potentiate the protective effects of native polysaccharide vaccines in humans.

Co-stimulation of B cells via the BCR and TLRs provides a potent, yet context-dependent, mechanism for modulating B cell activation, class-switch recombination, and Ab secretion. TLR and BCR signaling activate NF-kβ through distinct pathways and thus MyD88-dependent signaling may synergize with TI-2 Ag-induced BCR crosslinking to support proliferation, isotype switching, survival, and differentiation into both memory B cells and long-lived ASCs^36^, especially in B-1 and MZ B cells^37^, which typically contribute to TI-2 responses. In particular, previous work with mouse B cells has demonstrated that BCR and TLR signaling promote enhanced CSR —for example, anti-IgD–dextran plus TLR1/2, 4, 7, or 9 ligands in the presence of IL-4 induced IgG1 CSR via NF-κB–dependent upregulation of AID^36^. Although this approach stimulates extensive crosslinking, similar to TI-2 Ags with repeating epitopes, both IgM and IgD crosslinking would be expected to occur with a TI-2 Ag. Indeed, innate B cells, which are largely responsible for producing Ab in response to TI-2 Ags, regardless of whether the TLR4-based adjuvant is used^4^, express high levels of IgM and low levels of IgD. Solely crosslinking IgD versus both isotypes in these types of studies may skew towards follicular B cell responses and may therefore not adequately model TI-2 Ag-mediated activation of innate B cells^38,39^. Further, given the limited availability of IL-4 during these responses, CSR to IgG1 is generally limited, with IgG3, and to a lesser extent, IgG2b being produced when MPL is used as an adjuvant for PPS^22^. Finally, IgD engagement restrains TLR-induced plasma cell differentiation by reducing BLIMP1 expression, consistent with the suppressive effect of prolonged BCR signaling on ASC formation^36^. The finding that delayed administration of TLR agonists by two days significantly increases Ab responses to PPS in mice^16–18^ supports the possibility that TLR costimulatory effects on TI-2 Ab responses may be most effective after the initial phase of strong, prolonged BCR crosslinking has occurred. It is therefore likely that formulations which extend TLR agonist in vivo half-life and/or potency, such as squalene-based emulsions as shown in our study, or liposomes^40^, contribute to the adjuvanticity of TLR4 agonists that are co-administered with polysaccharide Ags. Although work suggests physical co-engagement of the BCR and TLR synergizes to promote strong B cell activation^41,42^ and T cell-independent type 1 Ab responses when LPS or flagellin displaying multivalent epitopes are used^43^, the aforementioned studies support that TLR agonists need not be linked to TI-2 Ags to function as strong adjuvants. However, our work suggests non-conventional T cells may nonetheless be required to support IgG responses^24^.

Our work with MPL supports the idea that the costimulatory potential of TLR agonists for TI-2 Ab responses relies on B cell expression of TLRs. In contrast to mice, where several TLRs are expressed on naïve B cells, in humans, naïve B cells are reported to express low levels of TLR, whereas activated and memory B cells express TLR1/2, 2/6, 7, and 9^27,44^. Importantly, BCR engagement may upregulate TLR, as is the case for TLR9^44^. Indeed, human naïve B cell proliferative responsiveness to TLR1/2, 2/6, 7, and 9, but not TLR3 and 4, have been shown in the presence of soluble anti-Ig and T cell help^45^. Consistent with this, along with trends for increased proliferation, we observed significantly increased Ab production in purified B cell cultures when Pam3CSK4 (TLR1/2), FSL1(2/6), R848 (7/8), CpG (9), or adilipoline (2/7) was combined with multivalent BCR crosslinking and IL-2. While our focus led us to further study Pam3CSK4 as a suitable adjuvant for human polysaccharide vaccines, it certainly remains possible that TLR2/6, 7/8 or 9 agonists may also have potential for this purpose. Although we postulate Pam3CSK4 and other MyD88-activating TLR agonists exert their adjuvant effects by directly co-stimulating TI-2 Ag-activated B cells, as is the case for MPL, this remains to be formally tested. It is certainly possible that other mechanisms, including induction of IFN known to support these responses^4,46^, are also involved.

Important considerations for developing TLR agonists as vaccine adjuvants to co-stimulate Ag-activated B cells include the effect these agonists have on non-B cell populations and their interaction with other vaccine components—both of which may impact the protective Ab response. Indeed, the unexpected finding that combining TLR2 and TLR7 agonists (i.e., adilipoline or R837 plus Pam3CSK4) failed to induce PPS-specific IgG in contrast to when either agonist was used alone highlights the need for careful consideration of TLR agonist combinations. The factors contributing to this antagonism remain unknown. Conversely, Pam3CSK4 exhibited additive effects when combined with the MPL-based adjuvant in mice. Supporting this observation, there are documented instances of positive crosstalk between TLR2 and TLR4 in B cells and other cell types. Notably, TLR2 agonists have been shown to upregulate TLR4 expression on human B cells^47^, and the combined stimulation of TLR1/2 and TLR4 has been found to synergistically promote class switch recombination in mouse B cells^48^. Indeed, small amounts of contaminating TLR2 and TLR4 agonists in commercial pneumococcal vaccines have been found to support increased Ab responses in mice^49^. While the mechanism underlying Pam3CSK4-mediated rescue of MPL-based adjuvant activity in NHP is not yet known, it is possible that, in addition to directly co-stimulating Ag-activated B cells via TLR1/2, Pam3CSK4 may have also facilitated MPL activation of primate B cells by upregulating TLR4. Alternatively, Pam3CSK4 may have functioned independently of MPL in the vaccinated NHP. Indeed, the humanized mouse model demonstrated that Pam3CSK4-squalene emulsion was sufficient for potent adjuvant effects. While we have only addressed the capacity to boost polysaccharide-specific Ab responses in immunocompetent mouse models, future work will investigate the potential of this TLR-based adjuvant to promote long-lived Ab responses and durable, functional memory. Our work in mice thus far indicates that functional polysaccharide-specific B cell memory is indeed significantly increased with Pam3CSK4-based adjuvants.

One limitation of our study was that the effects of the Pam3CSK-based adjuvant on IgG responses in NHPs were more variable and, in some cases, less sustained than in mice. We nevertheless observed increases in primary IgM, IgG, and IgA responses in 2/3 to 3/3 animals for serotypes 4, 14, 19 F, and 23 F, and for serotype 3, increases in IgG (2/3) and IgA (3/3). Although Pneumovax23 and conjugate vaccines are not typically administered with boosting regimens in older adults, examining the capacity for adjuvant-driven boosting in NHP, as well as the extent to which these responses compare to conjugate vaccines, will be important, especially given our results in mice demonstrating that adjuvanted native polysaccharides can promote polysaccharide-specific IgG levels that are comparable to alum-adjuvanted conjugate vaccines in terms of quantity and possibly quality with respect to promoting enhanced opsonophagocytosis activity. It is also important to recognize the major difference in the anatomical distances between the injected muscle, lymphatic vessels, and draining lymph nodes in mice and humans/NHP. Significantly longer lymphatic transit distances likely influence the kinetics with which large polysaccharide Ags and small‑molecule TLR agonists reach antigen‑specific B cells. It is therefore possible that either slowing the transit of TLR agonists or physically linking the agonist to the polysaccharide may improve coordinated delivery. Finally, although Pam3CSK4-based adjuvant formulations promoted significantly increased Ab responses in both young adult male and female mice (Figs. 6–7) and by male and female human B cells (Fig. 5), the potential for sex‑ or age‑related differences in responsiveness remains to be determined.

In conclusion, our findings underscore the importance of rational adjuvant design tailored to the unique features of polysaccharide-specific B cell responses. Mechanistic work in mice has been critical for the field of adjuvant development. However, key differences in the ability of adjuvants optimized for protein-based vaccines to similarly function as adjuvants for polysaccharide vaccines have hindered progress. By identifying Pam3CSK4 as a potent MyD88-activating adjuvant that enhances IgG responses across species, we provide a compelling foundation for advancing next-generation polysaccharide vaccines. The ability to selectively harness synergistic TLR signaling while avoiding antagonistic combinations opens new avenues for the rapid development of vaccines for emerging pathogens, as well as for providing alternative vaccines, particularly for populations where current conjugate vaccines are limited by availability, cost, complexity, or immunogenicity. Ultimately, our findings lay the groundwork for a more versatile and mechanistically informed approach to eliciting durable, protective humoral immunity against encapsulated bacterial pathogens.

## Methods

This research complies with all relevant ethical regulations and is consistent with the Wake Forest University School of Medicine’s Institutional Animal Care and Use Committee and Biosafety Committee, which approved the study protocol.

### Study design

Based on our initial experiments focused on evaluating the mechanisms by which MPL-based adjuvants improve polysaccharide-specific Ab responses in mice (via B cell-dependent TLR4/MyD88 activation) and their observed lack of effect in NHP, our goal was to determine whether other MyD88-activating TLR agonists could serve as adjuvants for polysaccharide vaccines in mice, NHP, and humans. For the in vivo studies, we aimed for at least five mice per group, based on our previous work with adjuvant evaluation in mice^22^. We followed a similar approach in humanized mice, using five donors for reconstitution studies. AGM was leased with the goal of four animals per group due to cost constraints. Animal numbers are included in figure legends. In these studies, biological replicates are considered individual animals, or in the case of humanized mice, individual donor PBMC used to reconstitute an NSG mouse receiving a particular treatment. In vitro assays used one individual and unique donor on different days—each was considered an experimental and biological replicate. The number of times an experiment was repeated is indicated in the figure legend. For mouse experiments, we collected sera up to day 50 or 60, based on our previous work. However, we limited serum collection in humanized NSG mice to d28, given the potential for GVHD development. NHP sera were also only collected for primary responses, given the associated lease costs. Data from animals were included if they were healthy at baseline and throughout the vaccination regimen. Data from one AGM who had an infection and antibiotic treatment during the study were excluded. Data from humanized mice were included if engraftment was confirmed by circulating human Ab (total Ab) at day 21.

### Mice

Wild type (WT), muMt, TLR4^-/-^, MyD88^-/-^, Trif^-/-^, and B1-8^hi^ IgH knock-in (B6.129P2-Ptrpca Ightm1Mnz/J) mice on a C57BL/6 background were from the Jackson Laboratory and were bred in-house under SPF conditions. Mice were housed in an environment with 30–70% humidity and ambient temperature (68–74°F) with a standard 12 h light/12 h dark cycle. NSG (NOD.Cg-Prkdcscid Il2rgtm1Wjl/SzJ) mice were obtained from the Jackson Laboratory and were maintained on drinking water containing sulfamethoxazole (0.8 mg/ml) and trimethoprim (0.16 mg/ml). Mice were age- and sex-matched for studies. Adult female African Green Monkeys (AGM) were leased from Wake Forest Primate Center’s interventional cohort^34^. Studies were approved by Wake Forest University School of Medicine’s Animal Care and Use Committee.

### Immunizations and ELISAs

Mice were immunized with the equivalent of ~0.125 μg each PPS contained within Pneumovax23 (Merck) or 5 μg NP40-Ficoll (Biosearch Technologies) in 50 μl PBS intramuscularly (i.m.) unless otherwise indicated. Mice were administered Prevnar13 (Pfizer) i.m., containing the equivalent of ~0.125 μg each PPS. Sigma Adjuvant System containing 20 μg *Salmonella minnesota* MPL, 20 μg synthetic cord factor (trehalose-6,6‘-dicorynomycolate (TDCM) in 0.5% squalene/0.05% Tween-80 was mixed with Ags before injections. Alternatively, adjuvant containing agonist, including Pam3CSK4 (20 μg), R837 (20 μg), adilipoline (20 μg), flagellin, and/or TDB (20 μg) was mixed with 40 μl of the squalene oil in water formulation, Addavax (all from InVivogen). AGM were immunized i.m. (right quadricep) with Pneumovax23 containing ~12.5 μg each PPS in 250 μl saline (half the human dose) alone or with adjuvant (250 μl Sigma Adjuvant System containing 125 μg MPL and 125 μg TDCM and squalene oil in water emulsion) or Sigma Adjuvant System with 125 μg Pam3CSK4 added.

Total Pneumovax23- and PPS-specific ELISAs were performed using 10 μg/ml cell wall polysaccharide-Multi (SSI Diagnostica) to block cell wall-specific Ab binding prior to sera addition to 1% BSA-blocked PPS-coated (5 μg/ml) Nunc Maxisorp 96-well plates^24^. NP-specific ELISAs were performed using 5 μg/ml NP_25_-BSA as a coating antigen^1,24^. As extensively detailed in our previous publication^24^, PPS Ab concentrations were estimated with an indirect method that involves standard curves generated using goat anti-mouse Ig (H + L) or goat anti-human Ig (H + L) capture Abs in conjunction with a dilution series of mouse or human IgM and IgG isotype standards (Southern Biotechnology), respectively. Human Abs were detected using anti-human IgM, IgG, and IgA alkaline phosphatase conjugates (Southern Biotechnology), whereas AGM Abs were detected using anti-monkey IgM, IgG, and IgA Abs (Fitzgerald). Relative PPS-specific IgA levels in AGM were determined using a standard curve developed with a dilution series of positive control sera. Linear regression, or, when appropriate, polynomial regression, was used to estimate the concentration of Abs in test samples.

### Mouse B cell adoptive transfer experiments and bone marrow chimera generation

MuMt mice were reconstituted with purified B cells (EasySep pan-B cell selection kit, StemCell) with 3×10^7^ wild-type, TLR4^-/-^, or Trif^-/-^ spleen B cells i.v. and immunized with 5 ug NP_40_-Ficoll i.m. the following day. For bone marrow chimera (BM) generation, WT mice were lethally irradiated (950 rad) and reconstituted i.v. with BM from muMt mice mixed with WT or MyD88^-/-^ BM (80:20 ratio; 1 ×10^7^ BM cells)^22,24^. Sulfamethoxazole (0.8 mg/ml) and trimethoprim (0.16 mg/ml) were supplied in drinking water 1 week prior to and 2 weeks following irradiation. Mice were rested for 8 weeks before immunization. For memory experiments, resting CD43^-^ CD45.1^+^ V_H_B1-8 Tg spleen (10^7^; i.v.) and peritoneal cavity (10^6^; i.p.) B cells were transferred to WT (CD45.2^+^) recipients, followed by i.m. immunization with NP_40_-Ficoll (5 μg) alone or mixed with adjuvant on d1. Purity ( > 95%) was determined by CD19^+^B220^+^dual staining on flow cytometry, For secondary transfers, 5 weeks post immunization, splenic (3×10^7^) and peritoneal B cells (6×10^5^) purified by negative selection (Thy1.2, GR1, F4/80, DX5, CD11c, CD138; Dynal) were transferred into naïve WT recipients which were then immunized with PBS, Ag, or Ag mixed with adjuvant.

### Human PBMC and mouse splenocyte cultures/ELISPOTS

Human PBMCs from whole blood or bloody buffy coats (Zenbio, SER-BC-SDS) obtained from healthy donors, aged 21–42, were purified using Lymphoprep (1.077 density). Buffy coats were harvested and washed in PBS. Cells were cultured at 2 to 4 ×10^5^ per 96 well in cRPMI+10% FCS containing 50 μM beta-mercaptoethanol and 10 ng/ml rhuIL-2 (Peprotech). The following agonists were added to cultures, as indicated: Pam3CSK4 (2 μg/ml), FSL-1(1 μg/ml), flagellin (1 μg/ml), R837 (1 μg/ml), R848 (1 μg/ml), CpG2006 (2 μg/ml), Adilipoline (1 μg/ml), Addavax (1:500, equivalent to 0.01% squalene), biotinylated goat anti-human Ig (H + L) F(ab’)_2_ (1 μg/ml; Jackson Immunoresearch), and/or streptavidin (5 μg/ml). In some experiments, PBMC were labeled with CFDA, SE (CFSE, 1 μM; Invitrogen) prior to culture. B cells were purified from PBMC using Miltenyi’s CD19 positive selection kit according to the manufacturer’s instructions. Cultures were incubated for 5 days, and cells were harvested for flow cytometric analysis (described below) or further culture in ELISPOT plates. For ELISPOTs, 20 μl cells were harvested on day 5 and placed in a PVDF Immobulon P plate (MSIPS4510) coated with goat anti-human Ig (H + L) (5 μg/ml) and preblocked with 180 μl media. Plates were incubated for 18 h in a CO_2_ incubator at 37 °C and developed using anti-human IgM, IgG, and IgA alkaline phosphatase conjugates (Southern Biotechnology), followed by NBT/BCIP development. For mouse B cell cultures, splenocytes were isolated from V_H_B1-8 Tg mice and cultured alone or with NP-Ficoll (10 ng/ml), TLR agonists, or both for 5 days and NP-specific IgM and IgG were measured in supernatants by ELISA^24^.

### Flow cytometry

PBMC cultures were harvested in PBS containing 2% newborn calf serum with the addition of Countbright beads, incubated with normal mouse serum for 15 min, and stained with fluorochrome-conjugated Abs specific for CD20 (2H7), CD86 (Fun1), IgG (G18-145), IgM (MHM-88), CD11b (M1/70) as well as Live/Dead Aqua for 30 min at 4 °C. For V_H_B1-8 Tg memory B cell analysis, cells were stained with NP_40_-APC, and fluorochrome-labeled antibodies to CD19, B220, and memory markers (CD80, CD73, PDL2) and live/dead exclusion dye^1,23^. Fluorochrome-labeled isotype controls were used to determine background staining levels. Antibodies and dilutions used are listed in Supplemental Table 1. Cells were analyzed using a BD FortessaX20 cytometer (BD Biosciences) with FSC-A/FSC-H doublet exclusion. Data were analyzed using FlowJo analysis software (Tree Star).

### huPBMC NSG chimeras

NSG mice were injected with ~10^7^ freshly isolated donor huPBMC i.p. Mice were immunized the following day with Pneumovax23, Pam3CSK4 + Addavax, or both i.p., with sera collected at 7-day intervals.

### *Streptococcus pneumoniae* challenge and opsonophagocytosis assay (OPA)

Mice were infected i.n. with serotype 3 WU2 strain *S. pneumoniae* by distributing 40 μl bacteria (1 ×10^7^ CFU) between 2 nares of isofluorane-anaesthetized mice^22^. Systemic pneumococcal infections and passive serum protection experiments were carried out by transferring sera and 1000 CFU TIGR4 or 100 CFU WU2 *S. pneumoniae* i.p. (100 μl) into CD19^-/-^ mice^23^. Mice were monitored daily for signs of distress and humanely euthanized. OPAs were performed as previously described^29,30^, with minor modifications. *S. pneumoniae* was resuspended at 5×10^4^/ml in opsonization buffer (OB: PBS with Ca^++^/Mg^++^, 10% heat-inactivated FBS, and 0.1% w/v sterile type A gelatin) and incubated with mouse sera (0.5–5%) for 30 min. Thioglycolate-elicited primary mouse macrophages (5×10^4^ per 96-round bottom well) in OB were combined with bacteria alone or with 25% rabbit complement (Cedarlane) and incubated at 37 °C with shaking (200 rpm), followed by incubation on ice for 20 min and plating on 5% blood agar plates for CFU determination.

### Statistical analyses

Data are shown as means ± SEM, with individual data points shown in figures. Measurements were taken from distinct biological samples. Differences between sample means were assessed using Student’s t-test, Mann-Whitney, or one- or two-way ANOVA with post hoc analysis as indicated in the figure legends. Differences in survival were assessed using the Log Rank test. Statistical analysis was performed using GraphPad Prism (version 10).

### Reporting summary

Further information on research design is available in the Nature Portfolio Reporting Summary linked to this article.

## Supplementary information

Supplementary Information

Reporting summary

Transparent Peer Review file

## Source data

Source Data

## Data Availability

All data associated with this study are present in the paper or the Supplementary Materials. Source data are provided with this paper and data have also been deposited in Dataverse 10.7910/DVN/MRHTDB. Source data are provided with this paper.
