# Supplementary material for: Efficacy of Pam3CSK4 as a cross-species adjuvant for polysaccharide vaccines in humanized mouse and non-human primate models
Source: Nat Commun. 2026 Jun 12;17:7472. doi: 10.1038/s41467-026-74194-7 (PMC13408974; doi:10.1038/s41467-026-74194-7)
Supplement: Supplementary file 1 — Supplementary Information [file 41467_2026_74194_MOESM1_ESM.pdf]

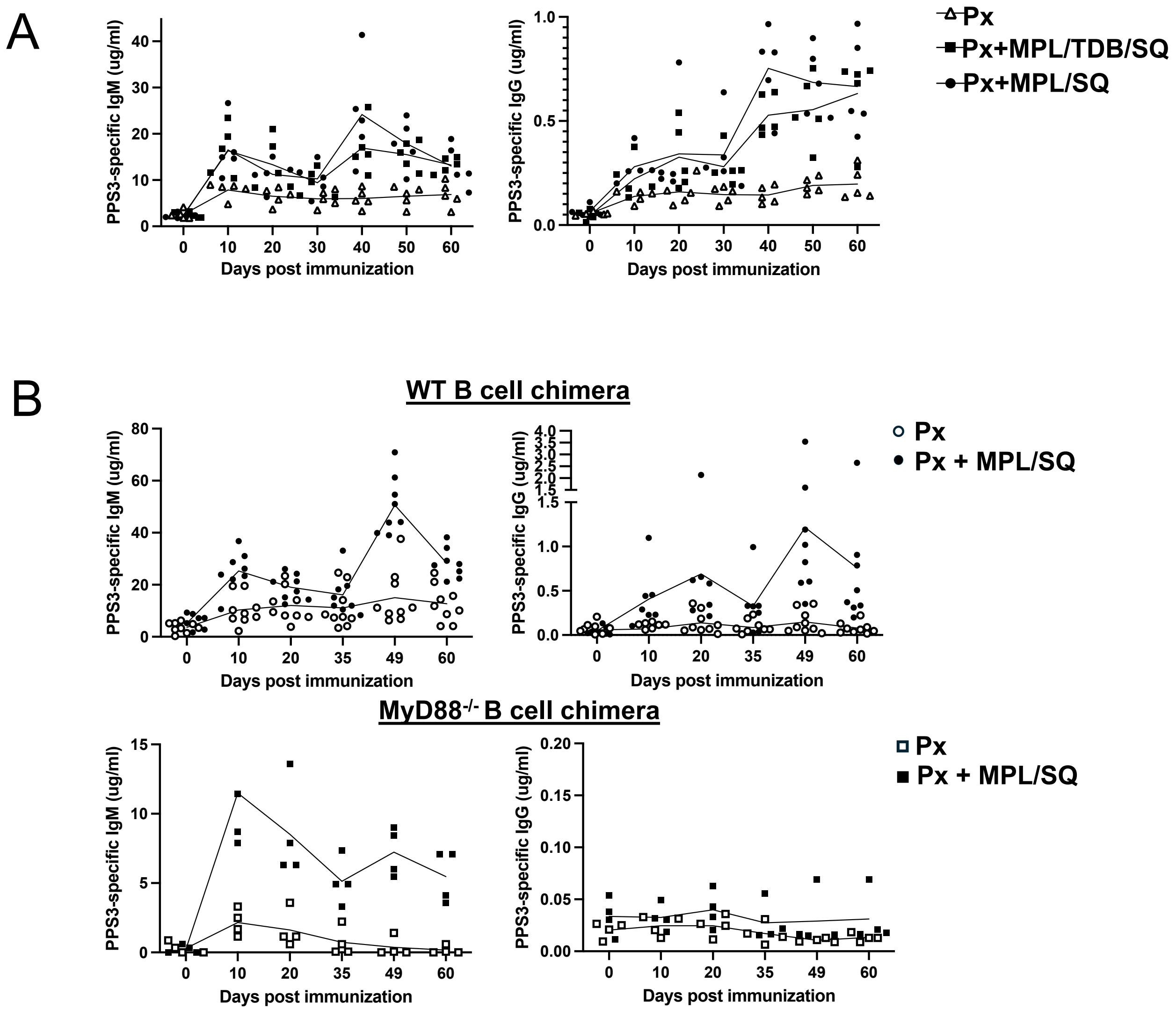

Supplemental Figure 1. MPL + squalene emulsion functions as an adjuvant for polysaccharide-specific Ab responses and requires MyD88 signaling on B cells (each symbol represents an individual mouse).

A) PPS3-specific IgM and IgG levels in C57BL/6 wild type (WT) male mice immunized with Pneumovax23 (Px) alone or combined with MPL + squalene emulsion (SQ), or MPL + TDB + SQ i.m., with a boost on d30 (n=5 mice/group). Mean values for these individual data points are shown in main Figure 1A. **B)** PPS3-specific IgM and IgG levels in male bone marrow chimeras reconstituted with male WT:muMt bone marrow or MyD88$^{-/-}$:muMt bone marrow (20:80), with n=8 WT B cell chimeras and n=4 MyD88$^{-/-}$ B cell chimeras per group. Mean values for these data are shown in main Figure 1C. In A-B, each symbol indicates a value obtained for an individual mouse.

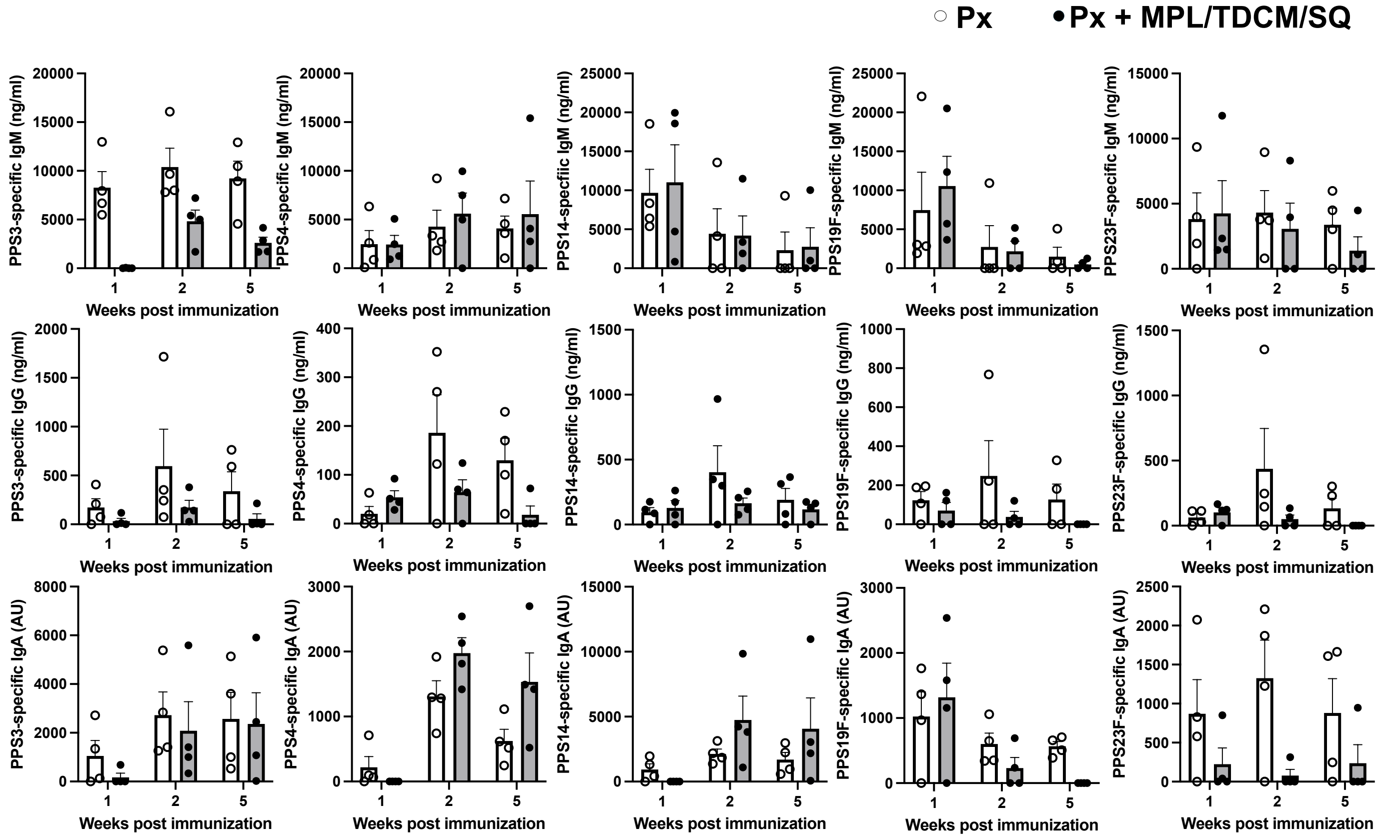

Supplemental Figure 2. MPL+ TDCM + squalene emulsion does not increase PPS3-, 4-, 14, 19F, or 23F-specific Ab responses in African Green monkeys (AGM).

PPS-specific IgM, IgG, and IgA responses in 17-20 year old female AGM 1, 2, and 5 weeks post-vaccination (i.m.) with Pneumovax23 (Px) containing 12.5 µg each PPS alone or mixed with MPL+TDCM+squalene emulsion (SQ) adjuvant. Individual baseline Ab values were subtracted from week 1, 2, and 5 Ab values to determine increases in PPS-specific IgM, IgG, and IgA levels over baseline as shown in graphs (n=4 AGM/group). Bar height indicates means with error bars indicating SEM. Circles indicate values for individual animals. No significant differences were found using a two-sided mixed effects model for analysis.

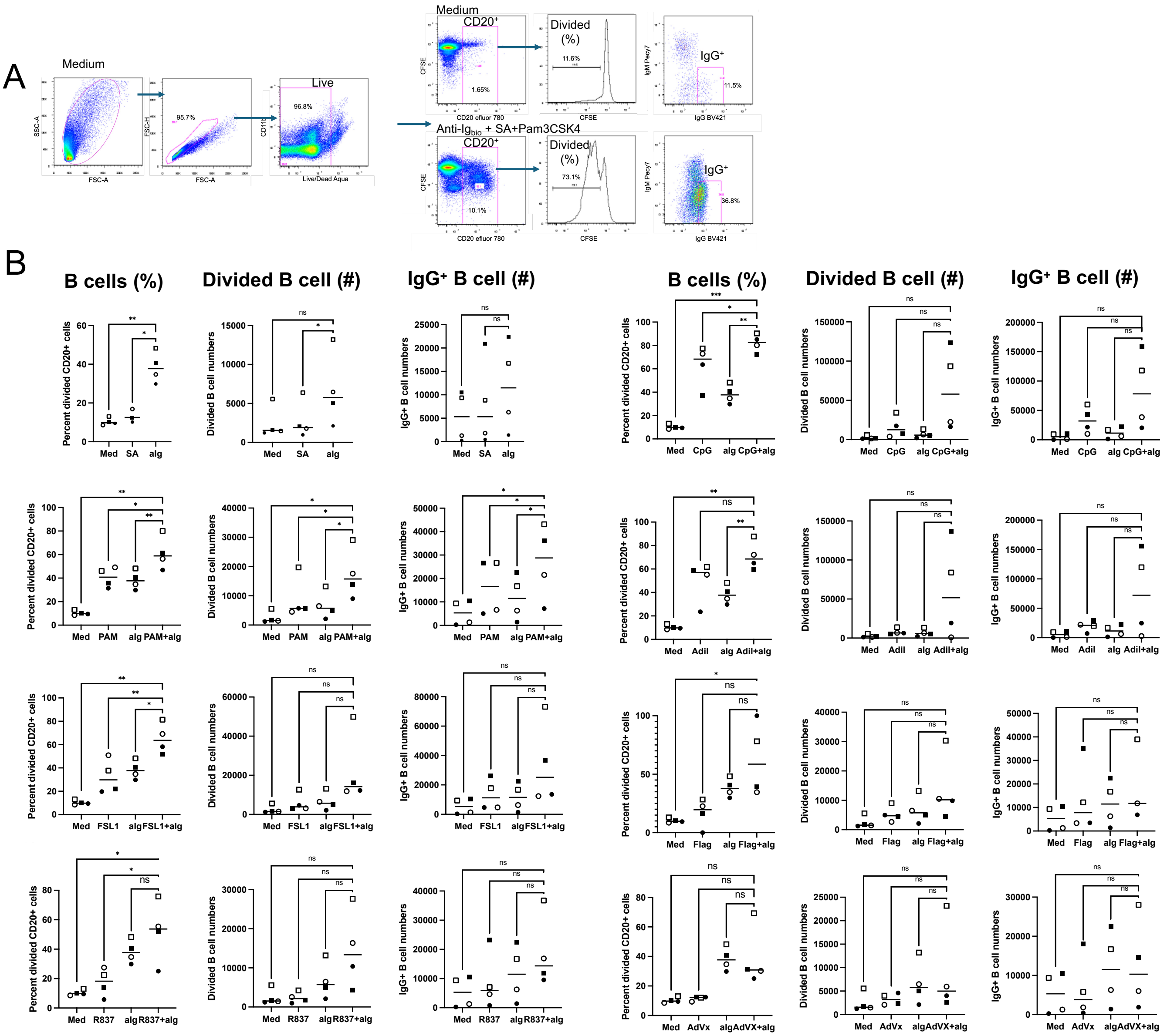

Supplemental Figure 3. Expansion of human B cells in PBMC with TLR agonists, biotinylated anti-Ig (H+L) + streptavidin, or both for 5 days.

A-B) PBMC from 4 human donors were labeled with 1 μM CFSE and cultured alone with rhuIL-2. TLR agonists, biotinylated anti-Ig (H+L) + streptavidin, or both for 5 days. Cells were harvested for flow cytometric analysis, and analyzed for viable CD20^{+} B cells, CFSE-divided B cell and IgG^{+} cell numbers (gating depicted in panel A). Data were analyzed by one way ANOVA with subsequent comparisons drawn for TLR agonist + BCR-activated B cells versus other conditions using Fisher's LSD. Asterisks indicate significant differences (*p<0.05, **p<0.01, ***p<0.001).

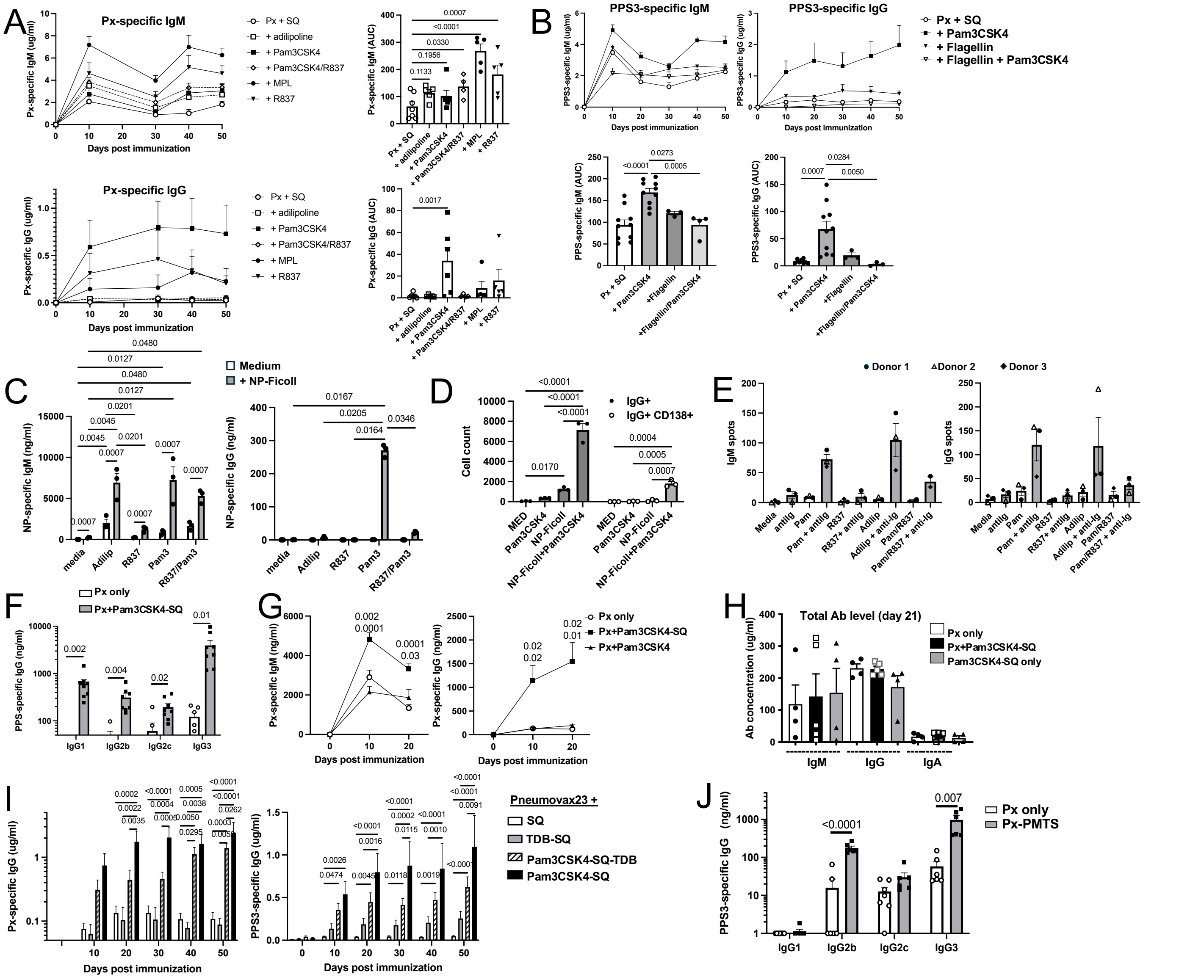

Supplemental Figure 4. Effects of adjuvants on PPS- and NP-Ficoll-specific antibody responses. A-B) Pneumovax23(Px) (A) or PPS3 (B)-specific IgM and IgG responses in WT male mice immunized i.m. with Px (0.125 µg each PPS) plus squalene emulsion (SQ) (40 µl) alone or combined with the indicated TLR agonists. Differences in responses between mice receiving TLR agonists versus mice immunized with Px+SQ alone were assessed by area under the curve (AUC) analysis and one-way ANOVA with Fisher's LSD two-sided post-hoc analysis in A: (Px+SQ, n=7; +Pam3CSK4, n=6; + Pam3CSK4/R837, n=4; all other groups n=5 mice/group) and in B: (Px+SQ, n=10; +Pam3CSK4, n=10; + flagellin, n=3; and +Pam3CSK4+flagellin, n=4). Symbols in AUC graphs indicate values for individual mice with means indicated by bar height and error bars representing SEM. **C-D)** V_HB1-8 Tg splenic B cells (from n=3 donors) were cultured with NP-Ficoll, adilipoline, Pam3CSK4, R837, Pam3CSK4 plus R837 individually or combined for 5 days. NP-specific IgM and IgG production was assessed in supernatants by ELISA (C) and NP-specific B cells were assessed for class switching and differentiation to ASC ($CD138^+$) by flow cytometry (D). Bar height indicates mean values with SEM indicated. Exact p values above bars indicate significant differences as assessed by one-way ANOVA with Bonferroni's multiple comparisons test (two-sided). **E)** Pam3CSK4-supported increases in anti-Ig-induced ASC in ELISPOT analysis in purified human B cell cultures (d6) were reduced when R837 was added (n=3 human donors/group). **F)** Total mean (±SEM) Px-specific IgG1, IgG2b, IgG2c, and IgG3 levels (d50) in mice immunized i.m. with Px alone (n=6 mice) or with Pam3CSK4-SQ (n=8 mice). Differences were assessed using two-tailed unpaired Student's t-test. **G)** PPS3-specific IgM and IgG levels in WT mice in response to Px (n=12), Px+Pam3CSK4 (n=6), Px+Pam3CSK4+SQ (n=10). Means (±SEM) are shown. P values are shown for two-sided ANOVA followed by Dunnett's multiple comparisons test, with top number indicating result for comparison of Px+Pam3CSK4+SQ to Px only and bottom number indicating result for comparison to Px+Pam3CSK4. **H)** Circulating human Ab levels (mean ± SEM) in NSG mice 3 weeks post reconstitution. Each symbol represents an individual donor. **I)** Px-and PPS3-specific IgG levels (mean ± SEM) in response to Px alone (n=12) or mixed with Pam3CSK4-SQ (n=8), TDB-SQ (n=6), or both (n=5). P values indicate significant differences as assessed by repeated measures ANOVA with Tukey's multiple comparisons test (two-sided). **J)** IgG isotypes produced to PPS3 in mice immunized with Px alone or Px + Pam-MPL/TDCM-SQ (d50; n=6 mice/group). Significant differences were determined using two tailed unpaired Student's t-test.

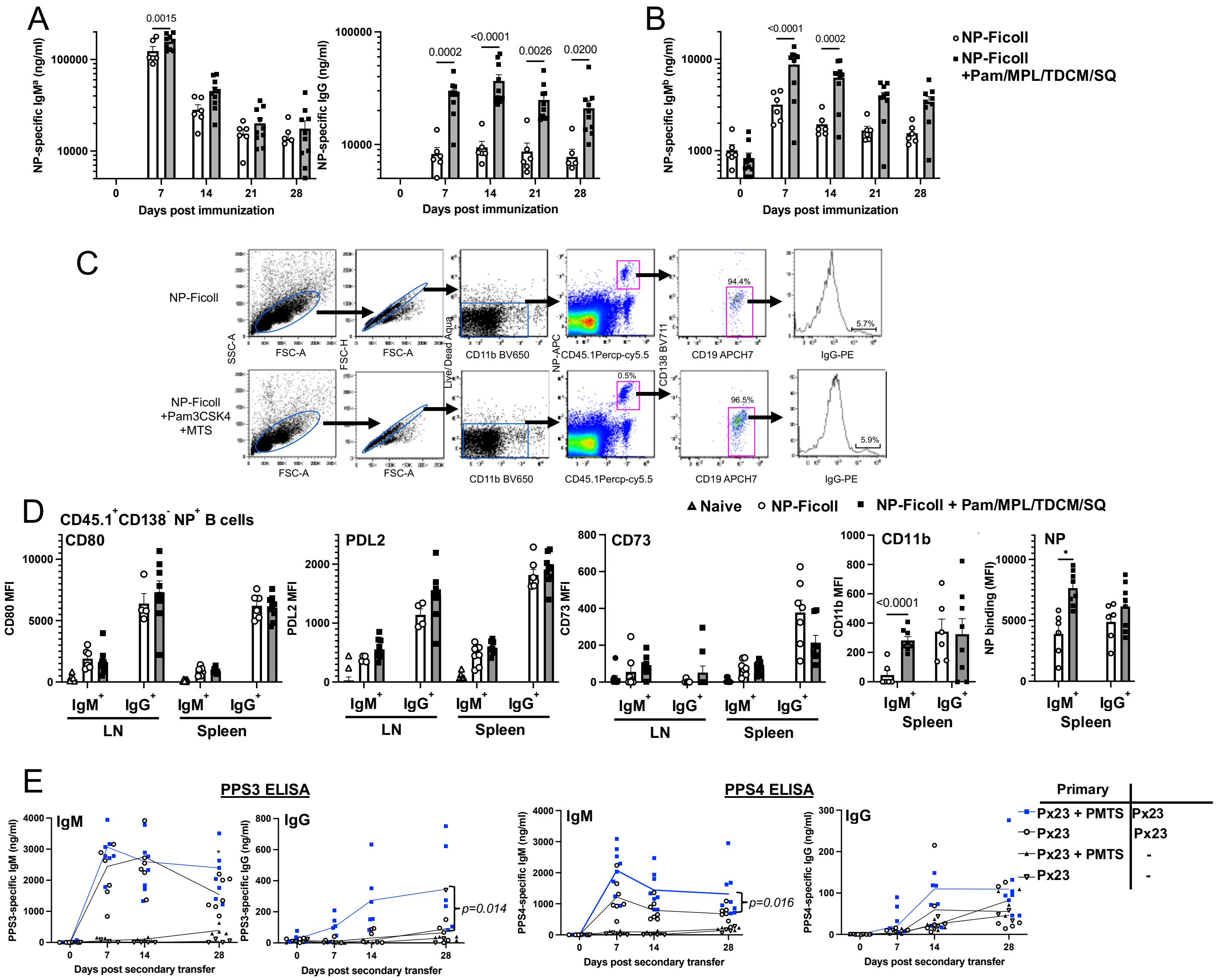

Supplemental Figure 5. Pam3CSK4-MPL/TDCM/SQ adjuvant increases generation of functional Ps-specific memory B cells.

WT mice were reconstituted with $CD43^{-}CD45.1^{+}V_{H}B1$-8 Tg B cells and immunized with 5 µg NP-Ficoll alone (n=6) or with Pam3CSK4-MPL/TDCM/SQ adjuvant (n=8) i.m. **A-B)** NP-specific IgM^{a} and IgG (A) and endogenous NP-specific IgM^{b} (B) was assessed in recipient mice. Values represent means ±SEM with p values shown indicating significance for the relevant comparisons as determined by two-sided repeated measures ANOVA with Bonferroni's multiple comparisons test. **C-D)** Five weeks later, splenic and draining lymph node $CD45.1^{+}CD138^{-}$ $CD19^{+}$ IgM^{+} and IgG^{+} NP-specific memory B cells (as depicted using the gating strategy in C) were assessed for expression levels of CD80, PDL2, CD73, CD11b, and NP binding. P values indicate significant differences as determined by two-tailed unpaired Student's t test. **E)** Splenic B cells from WT mice immunized with Pneumovax23 were adoptively transferred into naïve WT recipients. These recipients either remained naïve (n=4) or were immunized with Pneumovax23 i.m. (n=6), with PPS3- and PPS4-specific IgM and IgG responses assessed. P values indicate significant differences between Pneumovax23 secondary boost responses between mice primed with Pneumovax only versus Pneumovax + PMTS adjuvant as determined by two-sided repeated measures ANOVA. In A-E, data points for individual mice are shown.

Supplemental Table I. Antibodies used in flow cytometry

Mouse B cell staining antibodies

Antibody	Fluorochrome	Dilution	Company	Clone	Catalog #
Rat anti-mouse CD11b	BV650	1 to 1000	BD Biosciences	M1/70	563402
Anti-mouse CD73	BV605	1 to 600	Biolegend	TY/11.8	127215
Rat anti-mouse IgM	BV421	1 to 400	BD Biosciences	R6-60.2	562595
Rat anti-mouse CD138	BV711	1 to 500	BD Biosciences	281-2	563193
Rat anti-mouse CD19	APC-H7	1 to 500	BD Biosciences	1D3	560143
Live Dead Fixable Blue	BUV395	1 to 500	Invitrogen	-	L23105
Mouse anti-mouse CD45.1	PerCP-Cy5.5	1 to 500	BD Biosciences	A20	560580
Anti-mouse CD80	PE-Cy7	1 to 600	Biolegend	16-10A1	104734
Rat anti-mouse IgG1	PE	1 to 700	Southern Biotech	SB77e	1144-09
Goat F(ab')2 Anti-mouse IgG2b	PE	1 to 700	Southern Biotech	-	1092-09
Goat F(ab')2 Anti-mouse IgG3	PE	1 to 600	Southern Biotech	-	1102-09
NP40	APC	1 to 1500	Made 12-1-17 by KH	-	-
Rat Anti-mouse PDL2	BV510	1 to 400	BD Biosciences	TY25	740194
Rat IgG2a K	BV605	1 to 600	Biolegend	RTK2758	400540
Rat IgG2b K	BV421	1 to 400	Biolegend	RTK4530	400639
Rat IgG2a K	BV711	1 to 500	BD Biosciences	R35-95	563047
Rat IgG2b K	PE-Cy7	1 to 600	Invitrogen	eB149/10H5	25-4031-82
Rat IgG2b K	PE	1 to 700	Invitrogen	eB149/10H5	12-4031-82
Rat IgG2a K	BV510	1 to 400	BD Biosciences	R35-95	562952

Human B cell staining antibodies

Antibody	Fluorochrome	Dilution	Company	Clone	Catalog #
CD138	BV711	1/100	Biolegend	M115	356521
anti-hu IgG	BV421	1/100	BD Biosciences	G18-145	562581
CD20	Efluor780	1/100	eBiosciences	2H7	47-0209-42
IgM	Pecy5	1/200	Biolegend	MHM-88	314531
Rat anti-CD11b	BV650	1/1000	BD Biosciences	M1/70	563402
Live/Dead Aqua	Aqua	1/1000	InVitrogen	-	L34957
