# Supplementary material for: Efficacy of Pam3CSK4 as a cross-species adjuvant for polysaccharide vaccines in humanized mouse and non-human primate models
Source: Nat Commun. 2026 Jun 12;17:7472. doi: 10.1038/s41467-026-74194-7 (PMC13408974; doi:10.1038/s41467-026-74194-7)
Supplement: Supplementary file 3 — Transparent Peer Review file [file 41467_2026_74194_MOESM3_ESM.pdf]

nature portfolio

Peer Review File

Efficacy of Pam3CSK4 as a Cross-Species Adjuvant for Polysaccharide Vaccines in Humanized Mouse and Non-Human Primate Models

Corresponding Author: Dr Karen Haas

This file contains all reviewer reports in order by version, followed by all author rebuttals in order by version.

Version 0:

Reviewer comments:

Reviewer #1

(Remarks to the Author)
The study sought to evaluate the effect of an adjuvant mixture including MPL and squalene in induction of PS-specific antibody responses and protection against pneumococcal challenge in animals immunized with Pneumovax. Mice showed an improved response, which was dependent on TLR-4 MyD88 expressing B cells, while in monkeys the response was negligible.
Therefore, the authors investigated other MyD-88 dependent TLR agonists that could act as adjuvants for polysaccharide-based vaccines. The molecules were tested for co-stimulatory potential of B cells by flow cytometry and ELISPOT, and Pam3CSK4 (TLR1/2 agonist) was selected for yielding the most consistent results. Next, the selected adjuvants were tested in in vivo models of pneumococcal infection. In mice, the mixture of Pam3CSK4 and squalene provided protection against pneumococcal infection, while other combinations were not protective. A formulation containing Pam3CSK4 with MPL/TDCM and squalene emulsion was also shown to induce stronger antibody responses and protection in humanized mice, and to restore immune responses in monkeys.
The work provides a valuable contribution to the improvement of current polysaccharide-based vaccines and has great translational potential. The methods are clearly written and the experimental design is adequate.
The data fully supports the claims that TLR 1/2 agonists are promising adjuvants for polysaccharide vaccine formulations, inducing strong immune responses in different animal models, including humanized mice and non-human primates, as well as in human B cells.
Although the present data (specially the passive protection studies) supports that IgG production is important for protection against pneumococcal infection, it would be interesting to include opsonophagocytic assays to confirm the clearance mechanism. It would also be important to investigate the induction of memory to attest the duration of the observed responses, as mentioned in the discussion.
As a minor suggestion, in Line 95, rewrite this part of the sentence.

Reviewer #2

(Remarks to the Author)
Jennings-Gee et al. describe the lack of translatability of MPL’s ability to enhance immune responses to bacterial polysaccharide vaccines from mice to nonhuman primates and focus on identifying other adjuvants better suited for this purpose. They identify a few adjuvants using human immune cells ex vivo, and then through vaccine studies in mice show that PAM3CSK4 as an ideal candidate when combined with a squalene oil in water emulsion. The ability of PAM3CSK4 to enhance responses in NHPs was also confirmed. Overall, this is a well written manuscript but I found the figures quite hard to follow due to their size, lack of clear labels, especially considering the large amount of data presented. Also, although PAM3CSK4 when combined in a cocktail with other adjuvants does appear to enhance the immune responses in NHPs,

their impact on the important antigen-specific IgGs is less clear-cut.
I do have some comments to the authors that if included could improve the manuscript.
1) It would be interesting to see how the immune responses with their optimized formulation compare to an alum-adjuvanted CRM-conjugate vaccine such as Prevnar, ideally in NHPs. This will help determine whether PAM3CSK4's activity is sufficient to make unconjugated polysaccharide formulations a feasible alternative in the clinic.
2) As IgG titers in NHPs didn't seem to be enhanced greatly by the PAM3CSK4, could be useful for authors to evaluate responses after a booster dose with the same formulations to see how that impacts the IgG levels. The current data doesn't show a substantial improvement when adding 4 different adjuvants to pneumovax23, vs. just administering it alone in NHPs. I think discussing this limitation as well as how this would compare to conjugate vaccines would be important.
3) As mentioned above, a reworking of the figures to make them more clear, larger, with bigger font, etc. would be a great aid in helping the readers interpret and understand the data. Not sure if this is an instance of less is more, where removing some of the less critical data (or creating more supplemental figures) would help.
Minor comments:
Line 95: looks like authors weren't sure if to say "induced by" or "depended on" and included both by error
Line 100: 2nd M in muMt is usually capital, I believe
Line 109 and 114: Text mentions Figures 2A-2D, while actual figure has panels combined into 2 subpanels of 2A and B.
Line 126: looks like word is missing prior to "strong BCR…"
Line 174: Use of "alone" is misleading. To me it implies that these adjuvants were tested without the addavax. I would say single PRR agonists in combination with Addavax here. I understand they're trying to differentiate from the dual agonists further down, but have to be careful to not imply that these adjuvants were tested completely by themselves without the emulsion.
Line 377: replace "(refs)" with the appropriate refs.

Version 1:

Reviewer comments:

Reviewer #1

(Remarks to the Author)
The authors have successfully addressed all the concerns and suggestions raised in the previous round of review. The clarifications provided are satisfactory.

Reviewer #2

(Remarks to the Author)
With their revisions, the authors have sufficiently addressed my concerns.

Open Access This Peer Review File is licensed under a Creative Commons Attribution 4.0 International License, which permits use, sharing, adaptation, distribution and reproduction in any medium or format, as long as you give appropriate credit to the original author(s) and the source, provide a link to the Creative Commons license, and indicate if changes were

made.
In cases where reviewers are anonymous, credit should be given to 'Anonymous Referee' and the source.
The images or other third party material in this Peer Review File are included in the article’s Creative Commons license, unless indicated otherwise in a credit line to the material. If material is not included in the article’s Creative Commons license and your intended use is not permitted by statutory regulation or exceeds the permitted use, you will need to obtain permission directly from the copyright holder.
To view a copy of this license, visit https://creativecommons.org/licenses/by/4.0/

Response to reviewers
Jennings-Gee et al., *Nature Communications* manuscript

We are grateful for the thoughtful reviews and constructive suggestions provided by the reviewers. We appreciate their encouraging feedback. In response, we have added new data to address specific comments and have revised the figures and text to improve clarity. We believe these additions and edits have substantially strengthened the manuscript. Major changes are highlighted in yellow within the revised manuscript, and a detailed point-by-point response is provided below. The entirety of the review is included as italicized font. Reviewer comments are reproduced in italicized bold font, followed by our responses.

REVIEWER COMMENTS

Reviewer #1 (Remarks to the Author):

The study sought to evaluate the effect of an adjuvant mixture including MPL and squalene in induction of PS-specific antibody responses and protection against pneumococcal challenge in animals immunized with Pneumovax. Mice showed an improved response, which was dependent on TLR-4 MyD88 expressing B cells, while in monkeys the response was negligible. Therefore, the authors investigated other MyD-88 dependent TLR agonists that could act as adjuvants for polysaccharide-based vaccines. The molecules were tested for co-stimulatory potential of B cells by flow cytometry and ELISPOT, and Pam3CSK4 (TLR1/2 agonist) was selected for yielding the most consistent results. Next, the selected adjuvants were tested in in vivo models of pneumococcal infection. In mice, the mixture of Pam3CSK4 and squalene provided protection against pneumococcal infection, while other combinations were not protective. A formulation containing Pam3CSK4 with MPL/TDCM and squalene emulsion was also shown to induce stronger antibody responses and protection in humanized mice, and to restore immune responses in monkeys.

The work provides a valuable contribution to the improvement of current polysaccharide-based vaccines and has great translational potential. The methods are clearly written and the experimental design is adequate.

The data fully supports the claims that TLR 1/2 agonists are promising adjuvants for polysaccharide vaccine formulations, inducing strong immune responses in different animal models, including humanized mice and non-human primates, as well as in human B cells.

Although the present data (specially the passive protection studies) supports that IgG production is important for protection against pneumococcal infection, it would be interesting to include opsonophagocytic assays to confirm the clearance mechanism.

It would also be important to investigate the induction of memory to attest the duration of the observed responses, as mentioned in the discussion.

As a minor suggestion, in Line 95, rewrite this part of the sentence.

Authors' Response to Reviewer #1:

Comment: Although the present data (specially the passive protection studies) supports that IgG production is important for protection against pneumococcal infection, it would be interesting to include opsonophagocytic assays to confirm the clearance mechanism.

Response:
We thank the reviewer for this suggestion. In response, we performed opsonophagocytosis assays (OPA) to investigate mechanisms by which the adjuvant-elicited antibodies might facilitate bacterial clearance. We used a standard mouse macrophage OPA and performed the assay in the presence and absence of rabbit complement. With 0.5% serum, clearance was observed both in the presence and absence of complement for the adjuvanted immune serum (but not naïve serum); however, complement was required for the Pneumovax-only immune sera to elicit clearance (now Figure 6D). In the presence of a higher serum concentration (5%), both types of sera supported opsonophagocytosis in the absence of complement, but complement further increased bacterial clearance for the adjuvanted immune serum. This suggests that both Fc gamma- and complement receptor-mediated opsonophagocytosis may play a role in the increase protection observed with the adjuvanted sera in vivo. Consistent with this, the isotypes produced in response to Pam3CSK4-based adjuvants include both isotypes that engage Fc receptors and activate complement: IgM, IgG3 and IgG2b. These data are now shown in Supplemental Figures 4F (for overall Pneumovax-reactive IgG antibodies) and 4J (for PPS3-specific IgG isotypes). Again, we thank the reviewer for suggesting this experiment as we believe it enhances our understanding of the mechanisms by which this adjuvant may increase protection.

Comment: It would also be important to investigate the induction of memory to attest the duration of the observed responses, as mentioned in the discussion.

Response: We agree this is an important aspect of protection to address. In addition to evaluating boosting responses, we formally tested the induction of memory in two ways. First, we examined adjuvant effects in the V_HB1-8 Tg system. As shown now in Figure 8A, 5 weeks post adoptive transfer into WT mice, we examined NP-specific memory B cells in spleens and lymph nodes of recipient mice immunized with NP-Ficoll alone or with the combination adjuvant. The adjuvant resulted in 3-fold increases in splenic memory B cells, and 5- and 25-fold increases in IgM^+ and IgG^+ memory B cells in the draining lymph nodes, respectively. Transfer of the same number of spleen cells from recipient mice into new naïve recipients confirmed that the adjuvant resulted in significantly increased generation of memory B cells—this was evidenced by finding significantly increased recall (antibody) responses to unadjuvanted NP-Ficoll relative to memory cells that were generated with non-adjuvanted NP-Ficoll (now Figure 8B). We performed similar transfers of cells from mice immunized with Pneumovax with and without adjuvant, and then boosted all recipient mice with Pneumovax only. As shown in Figure 8C and Supplemental Figure 5E, recall responses to Pneumovax (non-adjuvanted) by memory cells transferred from mice that had received adjuvant during priming produced significantly increased PPS-specific IgM and IgG responses relative to recipients of donor cells from mice that had been immunized without adjuvant. These data support that the adjuvant promotes generation of memory cells that are functionally capable of responding to, and producing antibody against, non-adjuvanted antigen on secondary exposure. While we acknowledge the need for further study to investigate memory B cell durability and quality, these data support the potential for alternative strategies to induce functional Ps-specific B cell memory.

Minor comments: As a minor suggestion, in Line 95, rewrite this part of the sentence.

Response: We appreciate the reviewer pointing this out. We have revised this.

Reviewer #2 (Remarks to the Author):

Jennings-Gee et al. describe the lack of translatability of MPL's ability to enhance immune responses to bacterial polysaccharide vaccines from mice to nonhuman primates and focus on identifying other adjuvants better suited for this purpose. They identify a few adjuvants using human immune cells ex vivo, and then through vaccine studies in mice show that PAM3CSK4 as an ideal candidate when combined with a squalene oil in water emulsion. The ability of PAM3CSK4 to enhance responses in NHPs was also confirmed. Overall, this is a well written manuscript but I found the figures quite hard to follow due to their size, lack of clear labels, especially considering the large amount of data presented. Also, although PAM3CSK4 when combined in a cocktail with other adjuvants does appear to enhance the immune responses in NHPs, their impact on the important antigen-specific IgGs is less clear-cut.

I do have some comments to the authors that if included could improve the manuscript.
1) It would be interesting to see how the immune responses with their optimized formulation compare to an alum-adjuvanted CRM-conjugate vaccine such as Prevnar, ideally in NHPs. This will help determine whether PAM3CSK4's activity is sufficient to make unconjugated polysaccharide formulations a feasible alternative in the clinic.

2) As IgG titers in NHPs didn't seem to be enhanced greatly by the PAM3CSK4, could be useful for authors to evaluate responses after a booster dose with the same formulations to see how that impacts the IgG levels. The current data doesn't show a substantial improvement when adding 4 different adjuvants to pneumovax23, vs. just administering it alone in NHPs. I think discussing this limitation as well as how this would compare to conjugate vaccines would be important.

3) As mentioned above, a reworking of the figures to make them more clear, larger, with bigger font, etc. would be a great aid in helping the readers interpret and understand the data. Not sure if this is an instance of less is more, where removing some of the less critical data (or creating more supplemental figures) would help.

Minor comments:
Line 95: looks like authors weren't sure if to say "induced by" or "depended on" and included both by error
Line 100: 2nd M in muMt is usually capital, I believe
Line 109 and 114: Text mentions Figures 2A-2D, while actual figure has panels combined into 2 subpanels of 2A and B.
Line 126: looks like word is missing prior to "strong BCR…"
Line 174: Use of "alone" is misleading. To me it implies that these adjuvants were tested without the addavax. I would say single PRR agonists in combination with Addavax here. I understand they're trying to differentiate from the dual agonists further down, but have to be careful to not imply that these adjuvants were tested completely by themselves without the emulsion.
Line 377: replace "(refs)" with the appropriate refs.

<u>Authors' Response to Reviewer #2:</u>

Comment 1: It would be interesting to see how the immune responses with their optimized formulation compare to an alum-adjuvanted CRM-conjugate vaccine such as Prevnar, ideally in NHPs. This will help determine whether PAM3CSK4's activity is sufficient to make unconjugated polysaccharide formulations a feasible alternative in the clinic.

Response: We agree this is a very important consideration. Unfortunately, due to the need to perform this experiment in age-matched animals, this experiment would require obtaining at least two new groups of

NHP (Pneumovax with adjuvant versus Prevnar) and preferably three (Pneumovax only). At this time, we do not have sufficient funds to perform this important experiment. However, we are seeking additional funding to address this exact question and to further understand the mechanisms of adjuvant action. To begin to address this, we compared antibody responses to Pam3CSK4/MPL/TDCM-squalene adjuvanted Pneumovax and alum-adjuvanted Prevnar in adult mice. As we now show in Figure 7, Pneumovax administered with this Pam3CSK4-based adjuvant resulted in ~10-fold greater PPS3-and PPS4-specific IgM responses than Prevnar13 immunization but there was no difference in the level of IgG produced. Thus, our results in mice demonstrate adjuvanted Pneumovax may have the potential to serve as a feasible alternative in the clinic. However, further optimization is likely required, along with a better understanding of the duration of protection that can be achieved.

Comment 2: As IgG titers in NHPs didn't seem to be enhanced greatly by the PAM3CSK4, could be useful for authors to evaluate responses after a booster dose with the same formulations to see how that impacts the IgG levels. The current data doesn't show a substantial improvement when adding 4 different adjuvants to pneumovax23, vs. just administering it alone in NHPs. I think discussing this limitation as well as how this would compare to conjugate vaccines would be important.

Response: We appreciate the reviewer's point regarding the variable IgG increases observed in NHPs with the PAM3CSK4-based adjuvant relative to the more potent effects observed in mice. Nonetheless, we did observe increases in IgM, IgG, and IgA for 3/3 or 2/3 animals for serotypes 4, 14, 19F, and 23F, and for serotype 3, increases in IgG (2/3) and IgA (3/3). In this case, differences in lymphatic architecture between rodents and larger primate species likely impacts outcomes. NHP (and human) lymphatic vessels span greater anatomical distances. Given the larger distances to transport both antigens and adjuvants to draining lymph nodes from muscle and the requirement for both the large polysaccharide antigen and small TLR agonists to reach the antigen-specific B cells, either simultaneously or in tandem, it is possible that altered formulations and delivery strategies aimed at addressing differences in antigen/adjuvant transport may improve overall responses.

We have expanded the Discussion to more clearly address these limitations and discuss future directions. Sharing these initial NHP study results will provide rationale and justification for such follow-up investigations.

Comment 3: As mentioned above, a reworking of the figures to make them more clear, larger, with bigger font, etc. would be a great aid in helping the readers interpret and understand the data.

Response: We apologize for the difficulty encountered in reviewing the figures in the first submission. The figures were embedded in the manuscript word file as smaller files, with resolution that may not have been preserved on submission. We have added more data and have included a significant amount of the data in the supplemental files. We are hopeful that the larger files, more detailed labeling, and supplemental data supplied with the revision allows for improved clarity and interpretation.

Minor comments:
Line 95: looks like authors weren't sure if to say "induced by" or "depended on" and included both by error

Line 100: 2nd M in muMt is usually capital, I believe

Line 109 and 114: Text mentions Figures 2A-2D, while actual figure has panels combined into 2 subpanels of 2A and B.

Line 126: looks like word is missing prior to "strong BCR…"

Line 174: Use of "alone" is misleading. To me it implies that these adjuvants were tested without the addavax. I would say single PRR agonists in combination with Addavax here. I understand they're trying to differentiate from the dual agonists further down, but have to be careful to not imply that these adjuvants were tested completely by themselves without the emulsion.

Line 377: replace "(refs)" with the appropriate refs.

Response: We thank the reviewer for pointing these errors out. We have corrected them and revised the wording for clarity.
